# Exposure to Atmospheric Particulate Matter-Bound Polycyclic Aromatic Hydrocarbons and Their Health Effects: A Review

**DOI:** 10.3390/ijerph18042177

**Published:** 2021-02-23

**Authors:** Lu Yang, Hao Zhang, Xuan Zhang, Wanli Xing, Yan Wang, Pengchu Bai, Lulu Zhang, Kazuichi Hayakawa, Akira Toriba, Ning Tang

**Affiliations:** 1 Graduate School of Medical Sciences, Kanazawa University, Kakuma-machi, Kanazawa 920-1192, Japan; veronicayl@stu.kanazawa-u.ac.jp (L.Y.); zhanghao@stu.kanazawa-u.ac.jp (H.Z.); zhangxuan@stu.kanazawa-u.ac.jp (X.Z.); xingwanli@stu.kanazawa-u.ac.jp (W.X.); wangyan@stu.kanazawa-u.ac.jp (Y.W.); baipengchu@stu.kanazawa-u.ac.jp (P.B.); 2 Institute of Nature and Environmental Technology, Kanazawa University, Kakuma-machi, Kanazawa 920-1192, Japan; zhang-lulu@se.kanazawa-u.ac.jp (L.Z.); hayakawa@p.kanazawa-u.ac.jp (K.H.); 3 School of Pharmaceutical Sciences, Nagasaki University, Bunkyo-machi, Nagasaki 852-8521, Japan; toriba@nagasaki-u.ac.jp; 4 Institute of Medical, Pharmaceutical and Health Sciences, Kanazawa University, Kakuma-machi, Kanazawa 920-1192, Japan

**Keywords:** particulate matter, polycyclic aromatic hydrocarbons, personal exposure, indoor, outdoor, health effects

## Abstract

Particulate matter (PM) is a major factor contributing to air quality deterioration that enters the atmosphere as a consequence of various natural and anthropogenic activities. In PM, polycyclic aromatic hydrocarbons (PAHs) represent a class of organic chemicals with at least two aromatic rings that are mainly directly emitted via the incomplete combustion of various organic materials. Numerous toxicological and epidemiological studies have proven adverse links between exposure to particulate matter-bound (PM-bound) PAHs and human health due to their carcinogenicity and mutagenicity. Among human exposure routes, inhalation is the main pathway regarding PM-bound PAHs in the atmosphere. Moreover, the concentrations of PM-bound PAHs differ among people, microenvironments and areas. Hence, understanding the behaviour of PM-bound PAHs in the atmosphere is crucial. However, because current techniques hardly monitor PAHs in real-time, timely feedback on PAHs including the characteristics of their concentration and composition, is not obtained via real-time analysis methods. Therefore, in this review, we summarize personal exposure, and indoor and outdoor PM-bound PAH concentrations for different participants, spaces, and cities worldwide in recent years. The main aims are to clarify the characteristics of PM-bound PAHs under different exposure conditions, in addition to the health effects and assessment methods of PAHs.

## 1. Introduction

Air pollution has become a mainstream global environmental pollution problem in recent decades [1,2]. Particulate matter (PM) is one of the major factors contributing to air quality deterioration, leading to adverse health effects on humans [3,4,5,6]. PM is an extremely complex mixture defined in many ways, including formation pathway, emission source, chemical composition, and PM size [5]. The formation pathway of PM involves the direct release by emission sources into the atmosphere or secondary formation via nucleation, vapour condensation, adsorption, and absorption of gaseous precursors, primary PM, or secondary PM [5]. In terms of the source, natural sources include dust, sea salt, living vegetation, volcanic activity, and forest fires, whereas anthropogenic sources mainly involve combustion processes, including stationary sources (domestic, industrial, and agricultural activities) and mobile sources (vehicles, aircraft, and shipping traffic exhaust) [7,8]. Regarding the chemical composition, PM contains many kinds of inorganic and organic compounds, including water-soluble ions, trace elements, crustal material, elemental carbon, and organic carbon, many of which are harmful to human health [9,10,11,12,13]. According to the size, PM is generally divided into inhalable coarse particles (PM with an aerodynamic diameter smaller than 10 μm, PM_10_) and inhalable fine particles (PM with an aerodynamic diameter smaller than 2.5 μm, PM_2.5_) [14]. However, other sizes of PM also can be collected in the atmosphere by using different types of air sampler, such as PM_1_, PM_2_, PM_2.1_, and PM_4_, which aerodynamic diameters are smaller than 1, 2, 2.1, and 4 μm, respectively [15,16,17,18].

PM is of concern not only to researchers but also to the general public. Numerous toxicological and epidemiological studies have proven the adverse links between exposure to PM and health effects [19,20,21,22,23,24,25,26]. The cancer risk resulting from PM exposure has also been demonstrated by the International Agency for Research on Cancer (IARC) [27]. According to the report by the World Health Organization (WHO), PM in outdoor air is responsible for approximately 4.5 million premature deaths every year, or close to 10% of the total deaths on a global scale [28]. Among these deaths, approximately 2 million deaths, which represent approximately 5% of the global total deaths, are due to damage to the lungs and respiratory system directly attributable to PM [28]. Moreover, WHO also reported that almost 3 billion people worldwide still rely on solid fuels for cooking and heating, leading to approximately 4 million people premature deaths due to household indoor air pollution [29], which is almost equal to the deaths caused by outdoor PM pollution.

To protect public health, relatively strict indoor and outdoor air quality standards have been prescribed by WHO. For indoor household fuel combustion, WHO has strongly recommended that the emission rate target of PM_2.5_ should not exceed 0.23 mg/min under unvented conditions, and 0.80 mg/min under vented condition [29]. For outdoor air, the annual average PM_2.5_ and PM_10_ concentrations cannot exceed 10 and 20 μg/m^3^, respectively, and 24-h average concentrations that cannot exceed 25 and 50 μg/m^3^, respectively [30]. Currently, outdoor PM regulation standards also have been implemented in many cities worldwide. Table 1 lists various PM_2.5_ and PM_10_ regulation standards set by the governments of several countries according to their national conditions.

Specifically, in North America, the 24-h and annual average PM_2.5_ concentration standards in the United States of America (USA) are 35 μg/m^3^ and 12 μg/m^3^, respectively [3]. Mexico (12 μg/m^3^) and Canada (8.8 μg/m^3^) have also defined relatively low standards for the annual average PM_2.5_ concentration [31,32]. In South America, Brazil determined the final standards of PM_2.5_ and PM_10_ in 2018, which 24-h and annual average are the same as those prescribed by WHO [33]. However, Chile had relatively higher standards of PM_2.5_ (24-h: 50 μg/m^3^; annual: 20 μg/m^3^) than these above countries [34]. In Australia, the 24-h average PM_2.5_ and PM_10_ concentrations are the same as ones prescribed by the WHO, while the annual average of PM_2.5_ (8 μg/m^3^) is lower and of PM_10_ (25 μg/m^3^) is higher than the WHO-prescribed levels [35]. In the European Union (EU), Russia, and some Asian countries, the PM_2.5_ and PM_10_ regulation standards are mostly higher than those in the above countries. The annual average PM_2.5_ concentration standards are 15 μg/m^3^ (Japan), 25 μg/m^3^ (EU, Russia, South Korea, and Mongolia), 35 μg/m^3^ (China), and 40 μg/m^3^ (India), respectively [36,37,38]. In South Africa, only the PM_10_ regulation standard has been defined, which does not exceed 40 μg/m^3^ for the annual average concentration and 75 μg/m^3^ for the 24-h average concentration [39].

In PM, polycyclic aromatic hydrocarbons (PAHs) are a class of persistent organic chemicals with at least two aromatic rings, mainly directly emitted as a result of the incomplete combustion of various organic materials, including both natural sources, such as forest fires and volcanic eruptions, and anthropogenic sources, such as the combustion of fossil fuels and biomass [40,41]. Several hundred PAHs have been detected worldwide, and the United States Environmental Protection Agency (US EPA) has classified 16 PAH species in a priority control pollutant list. Table 2 lists the information of these 16 PAHs and certain non-priority PAHs, which can be generally divided into low-molecular-weight PAHs (LMW PAHs, MW < 200 g/mol), medium-molecular-weight PAHs (MMW PAHs, 200 ≤ MW < 250 g/mol), and high-molecular-weight PAHs (HMW PAHs, MW ≥ 250 g/mol) these three categories. LMW PAHs exhibit a relatively high vapour pressure and easily occur in the gaseous phase, whereas HMW PAHs exhibit a much lower vapour pressure than that of LMW PAHs, and they mainly occur in the particle phase [42,43,44,45,46,47]. The vapour pressure of MMW PAHs is between those of LMW and HMW PAHs [47,48,49,50], suggesting that they may occur in both the gaseous and particle phases, and phase partitioning largely depends on factors such as meteorological conditions (their occurrence in the gaseous phase increases at a relatively high ambient temperature, and their occurrence in the particle phase increases at a relatively low ambient temperature) [48,51]. In contrast, a previous study has reported that the half-lives of PAHs range from a few hours to days, and the half-lives of MMW and HMW PAHs are longer than that of LMW PAHs [52], which indicates that particulate matter-bound (PM-bound) PAHs could be transported across long distances to other regions worldwide before attenuation [53,54,55,56,57].

PAHs are widely known for their carcinogenicity, mutagenicity and toxicity, and they pose a serious threat to human health [58,59]. A previous study has noted that with increasing MW, the carcinogenicity and acute toxicity of PAHs increase and decrease, respectively [60]. However, LMW and MMW PAHs can react with other gaseous air pollutants, such as ozone (O_3_, which is a strong oxidizing agent that can damage human lung function, thus threatening human health [61]) and NO_x_, to produce derivatives with a relatively low vapour pressure that more easily occur in the particle phase than their parent PAHs, and their mutagenicity and toxicity may be higher than those of the parent PAHs [62,63]. Table 3 summarizes the evaluation of PAHs (including heterocyclic PAHs) and their derivatives by the IARC [27,64,65]. In addition to benzo[*a*]pyrene (BaP), which is classified in Group 1 (carcinogenic to humans), seven species are classified in Group 2A (probably carcinogenic to humans), and twenty-five species are classified in Group 2B (possibly carcinogenic to humans). Moreover, several emission sources in outdoor air, including coal combustion, coal tar pitch, coke production, diesel engine exhaust, tobacco smoke, and wood dust, are classified in Group 1 by the IARC [27], which may release many PAHs and derivatives. Due to these harmful effects on human health, it is necessary to clarify the concentrations, compositions, and major contributors of PAHs in the atmosphere.

In contrast to PM observations, real-time observations of PAHs have not been commonly analysed. According to Li et al. [66] real-time observation of PM can offer a high temporal resolution, but low specificity of chemical characterization. Amador-Muñoz et al. [67] performed real-time PAHs sampling in parallel to non-real-time PM sampling. The non-real time concentrations of PAHs in PM were all lower than the results from real-time PAHs sampling, possibly because PAH derivatives and heterocyclic species were also detected as PAHs when using the real-time method, and the PAHs concentration in the atmosphere is overestimated [67]. On the other hand, the techniques with high temporal resolution and high specificity for the chemicals in PM are very expensive for routine air quality monitoring [68]. Therefore, the non-real-time determination method is still a well-recognized method to determine the PAHs, including sampling, experimental treatment, and instrumental analysis. However, although the non-real-time observation method can measure different PAH species as needed, sample processing is a time-consuming task, the information of PAHs such as the atmospheric concentration cannot be promptly provided. To clarify the characteristics of the PAH species in PM, in our previous review, we summarized the size distributions of PM-bound PAHs freshly released from combustion sources and the distribution patterns of PM-bound PAHs in the atmosphere [69]. It was found that PAHs released from stationary sources are mainly bound to fine particles, but the sizes were slightly larger than that of PAHs released from mobile sources. In the atmosphere, PM-bound PAHs are more likely to be bound to large particles than to initial-mode particles, and the size decreases with increasing PAH MW [69]. Because the concentrations of PM-bound PAHs may differ according to people, the microenvironment, and area, in this review, we summarize the personal exposure concentrations of PM-bound PAHs and indoor and outdoor PM-bound PAHs in cities worldwide in recent years to further examine the exposure routes to atmospheric PM-bound PAHs and their health effects.

## 2. Concentrations of Atmospheric PM-Bound PAHs

### 2.1. Personal Exposure to PM-Bound PAHs

Research data have indicated that several factors, including conditions of the body, exposure routes, and environmental conditions, can influence the mechanisms of PAHs that are absorbed or adsorbed by human bodies [70]. PAHs may affect human health via inhalation, ingestion, and dermal (skin) exposure, with inhalation being the main exposure pathway for PM-bound PAHs [70]. Exposure varies from person to person due to variations in various physical factors such as the breathing rate. Table 4 lists several studies of personal exposure to PM-bound PAHs in different cities, which have focused on different participants, including non-occupational and occupational exposure.

Regarding non-occupational exposure, residents living in rural areas [71,72] generally inhale higher PM-bound PAH concentrations (4.2–655 ng/m^3^) than residents living in urban areas (0.4–11.9 ng/m^3^) [73,74]. This is because solid fuels such as coal and wood are still mainly used by rural residents for cooking and heating an many PAHs are emitted from these sources due to their low combustion efficiency, while urban residents mostly use clean fuels such as liquefied petroleum gas and natural gas. Thus, children living in Italy exhibited very low exposure levels and slight seasonal variation (0.65 and 0.63 ng/m^3^) [75], whereas children living in China exhibited relatively high exposure levels and a relatively large seasonal variation (27.31 and 58.18 ng/m^3^) [76]. A possible reason is the different urban type, whereby Rome (Italy) is a typical commercial city and Tianjin is one of the largest industrial centers of China, leading to the background concentration of PM-bound PAHs in Tianjin being higher than that in Rome [75,76]. In addition, the heating systems in Tianjin can lead to more PM-bound PAHs being released into the atmosphere in the winter [76]. Harbin is a large industrial city in northeast China, and it has been reported that the annual average PM-bound PAH exposure concentration in patients with chronic obstructive pulmonary disease is 186.85 ng/m^3^ [77], which is much higher than that seen in residents living in Hong Kong, Zhuhai, and Wuhan, China [73,74]. In Harbin, the annual temperature range can be up to 60 °C and the heating period can account for half of the year, thus, coal combustion for heating is also a large factor, in addition to industrial coal combustion, leading to the annual average concentration of PM-bound PAHs being higher than other cities in South and Central China [77].

Regarding occupational exposure, drivers, office workers, and policemen, as indicated in Table 4, did not exhibit very high PM-bound PAH exposure concentrations (1.03–39.08 ng/m^3^) but the values differed among cities and revealed seasonal variations [15,16,78]. In addition to the low background concentrations of PM-bound PAHs in cities, a possible reason is that few direct emission sources were impacting the above professionals in space. However, both housewives and highway toll station workers exhibited relatively high exposure levels [79,80,81], which were several times higher than those for the professionals described above. The high exposure levels for housewives (116 and 310 ng/m^3^) mainly occurred due to the use of fuel for cooking, while those for highway toll station workers (319.90 ng/m^3^) largely occurred due to the inhalation of traffic-related PAHs, such as vehicle exhaust [79,80,81]. Traffic emissions are also a major source for exposure for newsagents because their workplaces are usually located near roads. However, the exposure levels of PM-bound PAHs for newsagents were much higher in Tehran (Iran) (5570 ng/m^3^) [82] than those measured for highway toll station workers in Tianjin (China) [81]. A possible reason could be that the pollution levels may be higher in Tehran than that in Tianjin, and the background PM-bound PAH concentrations may be very high. Moreover, studies have revealed very high exposure levels of PM-bound PAHs for seafarers and Chinese kitchen workers, ranging from approximately 760 to 12,108 ng/m^3^ [83,84]. The high exposure levels for the former occur due to ship engine emissions and their long residence times on ships, while those for the latter occurred due to factors such as the use of various fuels, food type, cooking location and methods. Also, many PM-bound PAHs contained in food can be ingested by the human body through eating [85,86].

The results found that background concentrations in different cities could affect the exposure levels of residents, and the indoor microenvironment could also influence the exposure level because most people spend a lot of time indoors. In addition, the characteristics of the PAH phase distribution determine that the PM-bound PAH concentration is higher during cold periods than that during warm periods. Moreover, people with various jobs exhibit different exposure levels, especially regarding occupational exposure, and workers are exposed to very high health risks in spaces with high PM-bound PAH concentrations. Overall, although the PM type and determined PAH number differed among the above studies, personal exposure to PM-bound PAH depends on many complex factors, including indoor microenvironments, outdoor environments, daily activities (such as jobs), and body conditions.

### 2.2. Indoor Concentrations of PM-Bound PAHs

Indoor air quality is crucial to human health because people spend more than 80% of their time indoors [73]. The indoor PM-bound PAHs in different microenvironments are emitted by many sources, including cooking, smoking, heating, stoves, chemical spraying, machines such as laser printers, and outdoor sources. Studies have noted that indoor air may even be worse than the outdoor air with the PM-bound PAHs [71,87]. Table 5 summarizes the indoor concentrations of PM-bound PAHs in different microenvironments in certain cities in recent years. Some studies have simultaneously examined personal exposure, as described in Section 2.1 [71,73,79,80,83,84].

Regarding residential indoor air, the PM-bound PAH concentrations in rural households (738 ± 321 ng/m^3^) were much higher than those in urban households (0.186–276 ng/m^3^), and the concentrations observed in northern Chinese cities (15–276 ng/m^3^) were higher than those observed in southern Chinese cities (1.0–7.3 ng/m^3^) [71,73,79,80,88], similar to those of personal exposure (Section 2.1). The PM-bound PAH concentrations in households in Jeddah (Saudi Arabia) (18.5 ng/m^3^) [89] were comparable to those in Bursa (Turkey) (22 ng/m^3^) [90], while in Madrid (Spain) [91] they were much lower (0.186 ng/m^3^) than in the above cities. In both the Jeddah and Bursa studies air samplers were placed in the living room of residences, while in Madrid, Spain, it was placed on the first floor of a residential building [89,90,91]. The different locations of air samplers could lead to different PM-bound PAH concentration measurements. On the other hand, a high concentration of PM-bound PAHs (318 ± 314 ng/m^3^) was observed in infants’ rooms in Harbin (China) in winter, was not only suggested a high health risk for infants, but also for people who staying in the room [92].

Regarding school indoor air, Table 5 does not indicate a large concentration difference in Wuhan (China), among university dormitories (31.3 ng/m^3^), laboratories (27.0 ng/m^3^), and offices (32.4 ng/m^3^) [93]. The annual average concentration differences observed in universities between dormitories and offices in Beijing (34.1 and 32.1 ng/m^3^) were also not large [88], and comparable to those seen in Wuhan [93]. However, the concentrations observed in university offices in Jeddah (12.7 ± 5.1 ng/m^3^) [89] were lower than those observed in university offices in Beijing, consistent with the household results. Moreover, the PAH concentrations observed at the laboratories and offices in Harbin (115 and 96.6 ng/m^3^) in winter [94] were approximately three times higher than those observed in Beijing and Wuhan [88,93]. In contrast, the PM-bound PAH concentrations in primary or secondary classrooms ranged from 0.45 to 29.83 ng/m^3^, and the concentrations were comparable across Beijing, Warsaw and Gliwice (Poland), and Porto (Portugal), but they were very low in São Paulo (Brazil) [17,95,96,97]. Different from other sampling cities, São Paulo is close to the Equator and its hot climate can greatly influence the gaseous/particles phase distribution of PAHs. Moreover, the shorter sampling period used in São Paulo than in the other cities also had an impact on the PM-bound PAHs results [96].

Regarding public indoor air, the lowest concentration level (2.39–7.4 ng/m^3^) was observed in shopping malls in Islamabad (Pakistan), bakeries in Bari (Italy) and hotels in Jeddah [89,98,99]. This may occur because these public places contained few PAH sources. The second-highest concentration level (39.58–155.11 ng/m^3^) was observed in hotels in Jinan (China) in public bars in Warri (Nigeria) and in office buildings in Changchun (China) [100,101,102]. The higher concentration of PM-bound PAHs in hotels in Jinan than that in hotels in Jeddah possibly occurred due to the local background urban concentrations, and is consistent with the results for Beijing and Jeddah [88,89]. The relatively high concentration in public bars may be the result of factors such as tobacco smoking, while in office buildings, this may occur due to emissions from machines such as printers [103]. The seasonal differences observed in office buildings may occur due to the gaseous/particles phase distribution of PAHs at different temperatures and various outdoor pollution sources via window opening or ventilator operation [102]. The highest concentration level (550–39,000 ng/m^3^) was observed in fire stations, ships, and Chinese kitchens [83,84,104]. Smoke originating from fires contains a large number of PM-bound PAHs, which could be adsorbed onto the helmets and clothes of firefighters and transported to fire stations [104]. The high concentrations observed in ships and Chinese kitchens determine the personal exposure levels of seafarers and Chinese kitchen workers (Section 2.1), respectively, because of engine fuel combustion and cooking, respectively [83,84]. However, the lower concentration in kitchens than the personal exposure concentration for kitchen workers possibly occurred because the air sampler was likely not located close to the cooking bench for safety reasons (high temperature) [84].

In contrast to personal exposure studies, air samplers remain fixed indoors rather than being portable. Hence, the indoor concentrations of PM-bound PAHs are related to the type of space and sampler location. Different space uses to determine whether direct sources of PAH emissions occur, and different air sampler locations may determine whether more or less PAHs are collected. However, the air is exchanged between indoor and outdoor environments via opening windows or operating fans. Therefore, the air exchange speed and local outdoor concentration exert some considerable impacts on the indoor concentration. For example, if PM-bound PAHs are notably generated indoors and the air exchange speed is low, the indoor concentrations may be higher than the outdoor concentrations (such as in Chinese kitchens [84]). Although the PM type and determined PAH number differed among the above studies, the resulting impacts of PM-bound PAHs concentration levels were not large for the overall characteristics of different microenvironments, e.g., the concentrations were much higher in kitchens than those in classrooms, and higher in rural households than those in urban households.

### 2.3. Outdoor Concentrations of PM-Bound PAHs

The atmospheric behaviours of outdoor PM-bound PAHs are more complex because they are not only dependent on various direct emission sources including industrial and traffic emission [105], wood and biomass burning [59], but also many complex atmospheric physical and chemical factors, including interactions with other pollutants, photochemical degradation, and dry and wet deposition [106,107]. Due to the different conditions in countries worldwide, the concentrations of PM-bound PAHs also vary among regions. In this review, relevant reports (in English) were retrieved on outdoor PM-bound PAHs worldwide in recent years, and the available data are summarized in Table 6.

As indicated in Table 6, Auckland (New Zealand) attained the lowest annual average concentration of outdoor PM-bound PAHs (0.31 ± 0.19 ng/m^3^) [108] among all the cities/countries. Cities in the Americas also attained relatively low overall average concentrations levels of outdoor PM-bound PAHs, which ranged from 0.84 to 10.2 ng/m^3^ [67,109,110,111,112,113]. In Europe, the observations in A Coruña (Spain) (7.56 ng/m^3^) [114] exhibited a lower annual average concentration than that in cities in southern Spain (26.2 ng/m^3^) [115]. A seasonal variation was observed in Milan (Italy) (0.40 and 72.8 ng/m^3^) [116] and Wadowice (Poland) (10.5 and 80.6 ng/m^3^) [117] which was larger during cold periods than that during warm periods. During cold periods, the concentration of PM-bound PAHs in Nicosia (Cyprus) (1.62 ng/m^3^) [118] was the lowest among European cities. The concentrations observed in Brno (Czech Republic) (20.7 ng/m^3^) [119] were comparable to those observed in Zagreb (Croatia) (25.4 ng/m^3^) [120], whereas the concentrations in Sarajevo (Bosnia-Herzegovina) (64.8 ng/m^3^) [120] were much higher than those in Zagreb, even though the samples were collected during the same period. During warm periods, slight differences in the PM-bound PAH concentration were observed among Moscow (1.32–7.68 ng/m^3^), St. Petersburg (1.71–6.30 ng/m^3^), and Kazan (2.95–9.61 ng/m^3^) in Russia [121]. Although most countries in Europe as shown in Table 6 are developed countries, the overall concentration levels of outdoor PM-bound PAHs were relatively higher than those in the Americas. One reason was the different sampling periods that the concentrations were mostly annual average in the Americas, whereas were mostly seasonal average in Europe. In addition, different meteorological conditions in different periods can lead to the concentration differences of PM-bound PAHs. Moreover, the higher population density in Europe (~73/km^2^) than in the Americas (~21/km^2^), with more human activities including industrial and traffic emission also can increase PM-bound PAHs concentrations.

In Africa, the limited available studies involving short-term observations of outdoor PM-bound PAHs showed that the average concentrations in Algiers (Algeria) (7.47 ± 1.21 ng/m^3^) [122] and Pretoria (South Africa) (4.11 ng/m^3^) [123] were much lower than that in Kigali (Rwanda) (52.7 ng/m^3^) [124]. On the other hand, Ofori et al. [125] summarized the studies on the PAHs from 2005 to 2019 in Africa. Only 14 reports among 121 papers focused on the indoor and outdoor PM-bound PAHs. Of those, only four papers related to outdoor PM-bound PAHs in the last five years, in which air samples were collected in Rwanda (this report), Tunisia (urban area, 2.8 ± 3.4 ng/m^3^), Nigeria (industrial area, 73–143 ng/m^3^), and Egypt (rural: 323 ng/m^3^; suburban: 503 ng/m^3^; urban: 417 ng/m^3^) [125].

In Asia, China has the largest population globally. Since the severe haze event in 2013, researchers have paid special attention to the atmospheric environment. Yan et al. [126] reviewed 270 studies of PM-bound PAHs in 67 cities from 2001 to 2016, which covered seven typical regions, including North, Northwest, Northeast, East, Central, South, and Southwest China. It has been reported that the annual mean concentrations of PM-bound PAHs in these cities range from 3.35 to 910 ng/m^3^, and those in the northern regions are higher than those in the southern regions [126]. In this paper, Table 6 lists the concentrations of PM-bound PAHs in several major Chinese cities collected within 5 years. The results for these cities indicate average concentrations ranging from 1.36 to 1056 ng/m^3^ [18,73,127,128,129,130,131,132,133,134,135,136], and the seasonal and regional differences are consistent with the results reported by Yan et al. [126]. In other Asian cities, relatively low concentrations levels of PAHs have been observed in Japan [137,138,139], South Korea [140,141], Vietnam [142], Singapore [143], Malaysia [144], Thailand [145], Qatar [146], and Lebanon [147], ranging from 0.56 ng/m^3^ (Doha, Qatar) to 29.5 ng/m^3^ (Gwangju, South Korea). Relatively high PAH concentration levels have been observed in Mongolia [148], Pakistan [149], India [150,151,152], and Iran [153,154] ranging from 0.66 ng/m^3^ (Bushehr, Iran) to 773 ng/m^3^ (Ulaanbaatar, Mongolia), which countries are the most polluted areas in the world. In Mongolia, wood and biomass burning for cooking and heating were the largest emission sources of PM-bound PAHs in the winter [148], while in India and Iran, traffic emission was the main contributor for PM-bound PAHs through the years [151,153]. The air pollutants in Iran were also largely influenced by the air masses long-range transported from Iraq and other Middle East areas [155]. Moreover, due to the special location, frequent recirculation of air masses resulted in the increased residence time of PM in Iran and lead to high air pollutants levels [156].

According to the simultaneously obtained PM_2.5_ and PM_10_ data, as summarized in Table 6 [109,115,127,131,146,149,152], the concentrations of PM_2.5_-bound PAHs accounted for approximately 65%~95% of the total PM-bound PAHs, proving that PAHs mostly occur in PM with a small size, which is consistent with previous studies reporting that the adsorption of PAHs depends on the PM type [69]. Although high outdoor concentrations of PM-bound PAHs were observed in certain cities, they were not as high as the personal exposure concentrations for seafarers and kitchen workers nor the indoor concentrations observed in ships and Chinese kitchens [83,84]. This occurs because expansive outdoor spaces facilitate the diffusion and dilution of air pollutants. Moreover, outdoor air pollutants easily degrade or transform in the atmosphere due to various meteorological conditions and other reasons. Moreover, although there have been a large number of environmental observation studies on PM-bound PAHs over the past few decades, different countries and regions have focused on different PM-bound PAH research aspects. Some major cities have published many reports on various PAH species, while other cities have published no research reports on PAHs at all.

## 3. Health Effects and Assessments of PAHs

Over the last decades, many studies have been performed to better understand the health effects of PAHs. The health effects of PAH exposure can be divided into acute (short-term) effects and chronic (long-term) effects [59]. The acute effects mainly depend on the exposure time and PAH concentrations during exposure, while other factors, including pre-existing health conditions and age, may also influence health impacts [59]. Short-term exposure to high PAH levels may cause impaired lung function in patients with asthma and thrombotic effects in people suffering from coronary heart disease [157]. Acute occupational exposure to high PAH concentrations could cause eye irritation, nausea, vomiting, and diarrhea [158]. Repeated skin contacts with certain PAHs, such as anthracene (Ant) and naphthalene (NaP), are also known to cause skin irritation and inflammation [159]. However, PAHs to induce acute (short-term) human health effects at environmental concentrations are not fully understood.

In terms of chronic effects, long-term exposure to PAHs may induce DNA adduct formation in vitro and in vivo, in which the formation of DNA adducts is a key event regarding the mutagenicity and carcinogenicity of PAHs [160]. Rota et al. [161] summarized the studies of the respiratory and urinary tract cancers published between 1958 and 2014, that the high respiratory cancers (mainly lung cancer) were found in the workers who worked in iron and steel foundries for a long time. Petit et al. [162] investigated 93 exposure groups in nine industries and the results showed the highest lung cancer risk level was found in coke and silicon production, the lowest was in bitumen manufacture. Moreover, long-term exposure to PAHs can increase cardiovascular diseases (CVDs) risks and/or risk factors. Poursafa et al. [163] reviewed the related reports on exposure to PAHs and CVDs from 2000 to 2017. Most longitudinal with long-term follow-up studies indicated significant positive correlations of exposure to PAHs with CVDs increased risks. On the other hand, some studies reported that exposure to PAHs had negative effects on the development of children. For example, Kalantary et al. [164] summarized the studies of long-term exposure to PAHs with attention deficit hyperactivity disorder in children until 2018. Although overall studies did not show consistent results, the harm of exposure to PAHs on children is still worth noting and further research.

Regarding the assessment of PAHs, a large PAH database has been structured in various test systems including carcinogenicity, mutagenicity, and genotoxicity in the past decades [58,160]. Certain PAHs have been classified as carcinogenic to humans (Groups 1, 2A, and 2B, as indicated in Table 3) by IARC [27,64,65]. Several LMW PAHs, such as Ant and fluorene (Flu), have not been classified as carcinogenic. The carcinogenicities of some PAHs, such as acenaphthene (Ace), phenanthrene (Phe), and pyrene (Pyr), remain questionable [160]. On the other hand, because of the toxicity difference between different PAH species, their concentrations are insufficient to indicate the toxicity of PAHs. Hence, it is necessary to choose an appropriate index species for comparison. BaP is one of the most typical carcinogenic PAHs with the largest body of available data describing its exposure and health effects that BaP has commonly been adopted as the index species [160]. Table 7 summarizes the reference data of certain PAHs including toxicity equivalency factor (TEF) and relative potency factors (RPF) regarding their cancer risks based on BaP [160,165,166,167,168,169,170,171,172,173]. There is more available data for the 16 PAHs prioritized by the US EPA than for non-priority PAHs, and Table 7 indicates that among these 16 PAHs, the factor values are relatively low for most LMW PAHs and relatively high for most HMW PAHs. In addition to BaP, dibenz[*a,h*]anthracene (DBA) exhibits the highest factor value, which is 5 (TEF) and 10 (RPF) times higher than that reported by Nisbet and LaGoy [167] and the US EPA [160], respectively. Regarding the non-priority PAHs, certain PAHs exhibit relatively high factor values, such as benzo[*c*]fluorene (BcF), benz[*j*]aceanthrylene (BjA), and dibenzo[*a,l*]pyrene (DBalP), whose RPF values are 20, 60, and 30 times higher than that of BaP [160], respectively, suggesting that even if the concentrations of these species in the atmosphere are very low, their health effects on humans are extremely notable.

Currently, it is difficult to accurately assess the health effects of PAHs. The inhalation lifetime cancer risk (ILCR) model has been widely applied to estimate the health risk in people induced by inhalation exposure to PAHs [72], which is usually calculated according to two methods. One method is the occupational exposure assessment method, and the ILCR is calculated with Equation (1):

ILCR = BaP_eq_ × UR_BaP_ (1)

where BaP_eq_ is the BaP-equivalent concentration (ng/m^3^), calculated by multiplying the PAH concentration by TEF. UR_BaP_ is the unit cancer risk resulting from BaP, which is estimated as 8.7 × 10^−5^ per ng/m^3^, which is based on epidemiological data retrieved from studies on coke oven workers [58]. The other method is the non-occupational exposure assessment method, and the ILCR is calculated with Equation (2):

ILCR = BaP_eq_ × SF × IR× EF × ED × CF/(BW × AT) (2)

where SF is the cancer slope factor (mg·kg^−1^·day^−1^) for BaP inhalation exposure, IR is the inhalation rate (m^3^·day^−1^), EF is the exposure frequency (350 days·year^−1^), ED is the exposure duration (year), CF is the conversion factor with a value of 10^−6^, BW is the body weight (kg), and AT is the average lifespan for carcinogens (25,550 days). The population can be divided into males and females based on gender and subdivided into children, adolescents, adults, and senior adults based on age.

## 4. Conclusions

PM is one of the major factors contributing to air quality deterioration. PM regulation standards have been formulated in many countries, and the air quality index (AQI) of PM is monitored in real-time in most areas worldwide. Although PM-bound PAHs account for a very low percentage of PM, the behaviour of PAHs in the atmosphere is notable due to their carcinogenicity and mutagenicity. This review summarized several studies of personal exposure and indoor and outdoor PM-bound PAHs in recent years. The reviewed results indicated that personal exposure to PM-bound PAHs largely differed by the region, season, and job and depended on many complex factors. The indoor concentrations of PM-bound PAHs were related to the type of space use. The outdoor PM-bound PAH concentrations exhibited regional and temporal differences, and they were higher during cold periods than those during warm periods, while Asia was the region with the most serious PAH pollution. On the other hand, a novel coronavirus (COVID-19) was first discovered in China at the end of 2019, and after it broke out it then quickly spread all over the globe. To contain the COVID-19 epidemic, the outdoor activities of people (traffic, industry, recreation, etc.) were largely limited in many countries. Although the harm caused by COVID-19 is very large, several studies found that these control measures could greatly affect air pollutants emissions and the air quality, and the premature deaths due to improved air quality declined in many countries and regions [174,175,176,177], suggesting the control of air pollution should be more strengthened.

It has been more than four decades since the US EPA released a list of 16 critical PAHs. This list is known as the US EPA 16 PAHs, which has played a vital role in environmental and analytical research. However, there are several hundred PAH species globally, and some studies summarized in this review have analysed other PAH species, while certain non-priority PAHs exhibit higher toxicity than that of BaP. Furthermore, several PAH derivatives, such as oxygenated PAHs, nitrated PAHs, chlorinated PAHs (ClPAHs), and brominated PAHs (BrPAHs), are not included in the list, and ClPAHs and BrPAHs have only recently attracted attention. Toxicological studies have suggested that certain PAH derivatives exhibit even higher carcinogenicity and mutagenicity than those of their parent PAHs. For instance, 1-, 4-nitropyrene (1-, 4-NPs), and 6-nitrochrysene (6-NC) have been classified as Group 2A and 2B by IARC, which had higher TEF values (1-, 4-NPs: 0.1; 6-NC: 10) than Pyr and Chr [178]. These results indicate that the total health effects of PAHs are typically underestimated. A more effective measure of global health risk assessment of the exposure to PM-bound PAHs should be developed in the future.

## Figures and Tables

**Table 1:** Regulation standards of PM_2.5_ and PM_10_ set by several governments.

Continent	Country	PM_2.5_ (µg/m^3^)		PM_10_ (µg/m^3^)		References
		24-h	Annual	24-h	Annual	
North America	USA	35	12	150	None	[3]
	Mexico	45	12	75	40	[31]
	Canada	27	8.8	None	None	[32]
South America	Brazil	25	10	50	20	[33]
	Chile	50	20	150	50	[34]
Australia	Australia	25	8	50	25	[35]
Africa	South Africa	None	None	75	40	[39]
Europe	EU	None	25	50	40	[37]
	Russia	35	25	60	40	[38]
Asia	China	75	35	150	70	[36]
	Japan	35	15	None	100	[36]
	South Korea	25	25	100	50	[36]
	Mongolia	50	25	150	50	[36]
	India	60	40	100	60	[36]

**Table 2:** Information of US EPA 16 PAHs and non-priority PAHs.

Species (Abbreviation)	CAS Number	MW ^a^ Category	Vapor Pressure ^b^	Structure
US EPA 16 PAHs				
Naphthalene (Nap)	91-20-3	128.17 LMW	11.3	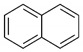
Acenaphthylene (Acy)	208-96-8	152.19 LMW	0.64	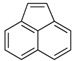
Acenaphthene (Ace)	83-32-9	154.21 LMW	0.29	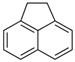
Fluorene (Flu)	86-73-7	166.22 LMW	0.08	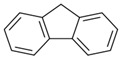
Anthracene (Ant)	120-12-7	178.23 LMW	0.08	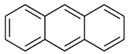
Phenanthrene (Phe)	85-01-8	178.23 LMW	1.61 × 10^−2^	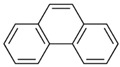
Fluoranthene (FR)	206-44-0	202.25 MMW	1.23 × 10^−3^	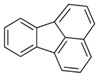
Pyrene (Pyr)	129-00-0	202.25 MMW	6.00 × 10^−4^	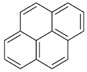
Benz[*a*]anthracene (BaA)	56-55-3	228.30 MMW	2.80 × 10^−4^	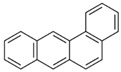
Chrysene (Chr)	218-01-9	228.30 MMW	8.31 × 10^−4^	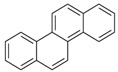
Benzo[*b*]fluoranthene (BbF)	205-99-2	252.30 HMW	6.67 × 10^−5^	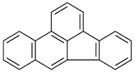
Benzo[*k*]fluoranthene (BkF)	207-08-9	252.30 HMW	1.29 × 10^−7^	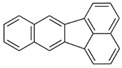
Benzo[*a*]pyrene (BaP)	50-32-8	252.30 HMW	7.32 × 10^−7^	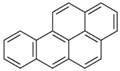
Dibenz[*a*,*h*]anthracene (DBA)	53-70-3	278.30 HMW	1.27 × 10^−7^	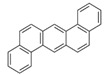
Indeno[1,2,3-*cd*]pyrene (IDP)	193-39-5	276.30 HMW	1.67 × 10^−8^	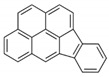
Benzo[*ghi*]perylene (BgPe)	191-24-2	276.30 HMW	1.33 × 10^−8^	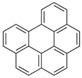
Non-priority PAHs				
Cyclopenta[def]phenanthrene (CdefP)	203-64-5	190.24 LMW	- ^c^	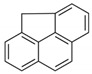
Benzo[*c*]fluorene (BcF)	205-12-9	216.28 MMW	- ^c^	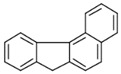
Cyclopenta[*c*,*d*]pyrene (CcdP)	27208-37-3	226.30 MMW	- ^c^	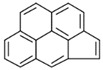
Benzo[*ghi*]fluoranthene (BghiF)	203-12-3	226.30, MMW	2.56 × 10^−5^	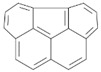
Triphenylene (Tri)	217-59-4	228.30 MMW	2.8× 10^−6^	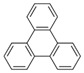
Benzo[*c*]phenanthrene (BcP)	195-19-7	228.30 MMW	- ^c^	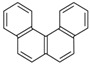
Retene (Ret)	483-65-8	234.30, MMW	- ^c^	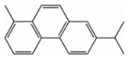
11H-Benz[*bc*]aceanthrylene (11H-BbcA)	202-94-8	240.30 MMW	- ^c^	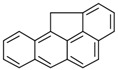
4H-Cyclopenta[def]chrysene (4H-CdefC)	202-98-2	240.30 MMW	- ^c^	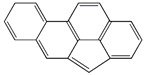
Benz[*j*]aceanthrylene (BjA)	202-33-5	252.30 HMW	- ^c^	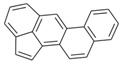
Benz[*e*]aceanthrylene (BeA)	199-54-2	252.30 HMW	- ^c^	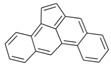
Benz[*l*]aceanthrylene (BlA)	211-91-6	252.30 HMW	- ^c^	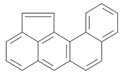
Perylene (Per)	198-55-0	252.30 HMW	6.67 × 10^−8^	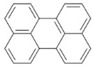
Benzo[*a*]fluoranthene (BaF)	203-33-8	252.30 HMW	- ^c^	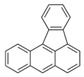
Benzo[*j*]fluoranthene (BjF)	205-82-3	252.30 HMW	3.60 × 10^−6^	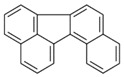
Benzo[*e*]pyrene (BeP)	192-97-2	252.30 HMW	7.60 × 10^−7^	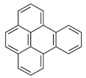
13H-Dibenzo[*a,h*]fluorene (13H-DahF)	239-85-0	266.3 HMW	- ^c^	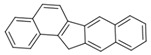
Anthanthrene (Anth)	191-26-4	276.30 HMW	- ^c^	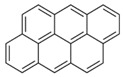
Indeno[1,2,3-*cd*]fluoranthene (IDF)	193-43-1	276.30 HMW	- ^c^	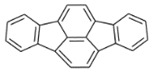
Benzo[*b*]chrysene (BbC)	214-17-5	278.30 HMW	- ^c^	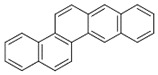
Benzo[*g*]chrysene (BgC)	196-78-1	278.30 HMW	3.07 × 10^−6^	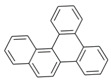
Benzo[*c*]chrysene (BcC)	194-69-4	278.30 HMW	1.20 × 10^−7^	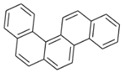
Dibenz[*a,c*]anthracene (DBacA)	215-58-7	278.30 HMW	1.33 × 10^−8^	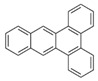
Dibenz[*a*,*j*]anthracene (DBajA)	224-41-9	278.30 HMW	- ^c^	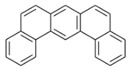
Picene (Pic)	213-46-7	278.30 HMW	- ^c^	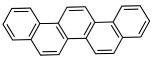
Coronene (Cor)	191-07-1	300.40 HMW	2.89 × 10^−10^	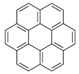
Benzo[*b*]perylene (BbPer)	197-70-6	302.40 HMW	- ^c^	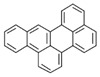
Naphtho[2,3-e]pyrene (NeP)	193-09-9	302.40 HMW	- ^c^	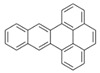
Dibenzo[*a*,*e*]fluoranthene (DBaeF)	5385-75-1	302.40 HMW	9.77 × 10^−9^	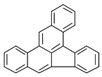
Dibenzo[*a*,*l*]pyrene (DBalF)	191-30-0	302.40 HMW	6.40 × 10^−8^	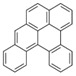
Dibenzo[*a*,*e*]pyrene (DBaeP)	192-65-4	302.40 HMW	6.93 × 10^−9^	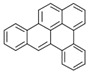
Dibenzo[*a*,*i*]pyrene (DBaiP)	189-55-9	302.40 HMW	2.40 × 10^−9^	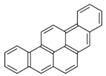
Dibenzo[*a*,*h*]pyrene (DBahP)	189-64-0	302.40 HMW	8.53 × 10^−10^	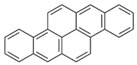

^a^: molecular weight (g/mol); ^b^: Pa at 25 °C. Data referred from Ambrose et al. [42], Coover et al. [43], Goldfarb et al. [44], Lee et al. [45], Lei et al. [46], Yamazaki et al. [48], Hoyer et al. [49], Sonnefeld et al. [50], and PubChem (National Library of Medicine) [47]; ^c^: No reference data.

**Table 3:** Evaluation of PAHs (including heterocyclic PAHs) and their derivatives by IARC.

Evaluation	Species
Group 1 ^a^	Benzo[*a*]pyrene
Group 2A ^b^	Dibenz[*a*,*h*]anthracene, Cyclopenta[*cd*]pyrene, Dibenzo[*a,l*]pyrene, Dibenzo[*c,h*]acridine, 1-Nitropyrene, 6-Nitrochrysene, 2-Nitrotoluene,
Group 2B ^c^	Naphthalene, Benz[*a*]anthracene, Chrysene, Benzo[*b*]fluoranthene, Benzo[*k*]fluoranthene, Benzo[*j*]fluoranthene, Indeno[1,2,3-*cd*]pyrene, Benzo[*c*]phenanthrene, Dibenzo[*a,e*]pyrene, Dibenzo[*a,h*]pyrene, Dibenzo[*a,i*]pyrene, Dibenzo[*a,h*]acridine, Dibenzo[*a,j*]acridine, Dibenzo[*c,g*]carbazole, 5-Methylchrysene, 2-Nitrofluorene, 4-Nitropyrene, 3,7-Dinitrofluoranthene, 3,9-Dinitrofluoranthene, 1,3-Dinitropyrene, 1,6-Dinitropyrene, 1,8-Dinitropyrene, 2,6-Dinitrotoluene, 3-Nitrobenzanthrone, 5-Nitroacenaphthene

^a^: Carcinogenic to humans; ^b^: probably carcinogenic to humans; ^c^: possibly carcinogenic to humans.

**Table 4:** Personal exposure concentration (average and/or range, ng/m^3^) of PM-bound PAHs in several studies.

Participants	PM	PAHs	Period	Concentration	Country, City
Rural residents	PM	28	July	655 ± 250	China, Laiyang [71]
Rural residents	PM_2.5_	10	9–12 March 2013	4.2–224	Thailand, Lampang [72]
Health residents	PM_2.5_	26	2014–2016	1.7 (0.4–5.2)	China, Hongkong [73]
Residents	PM_2.5_	16	2015–2018	8.27	China, Zhuhai [74]
Residents	PM_2.5_	16	2014–2017	11.9	China, Wuhan [74]
Children	PM_2.5_	8	11 April–9 May 2012 15 July–3 November 2012	0.65 0.63	Italy, Rome [75]
Children	PM_2.5_	16	17 May–23 June 2010 8 November–13 December 2010	27.31 58.18	China, Tianjin [76]
COPD patients	PM_2.5_	16	June 2017–October 2018	186.85	China, Harbin [77]
Drivers	PM_4_	12	2015–2018	9.97	Greece, Thessaloniki [15]
Office workers	PM_2_	13	2015	4.0 ± 2.3	Australia, Canberra [16]
Office workers	PM_2.5_	8	6–13 March 2009 10–19 June 2009	15.19 ± 15.15 3.04 ± 1.38	Czech Republic, Ostrava [78]
Policemen	PM_2.5_	8	8–20 February 2009 17–27 May 2009	4.27 ± 2.95 1.03 ± 0.61	Czech Republic, Prague [78]
Policemen	PM_2.5_	8	2–6 March 2009 6–10 June 2009	39.08 ± 17.33 4.27 ± 1.99	Czech Republic, Karvina [78]
Housewife	PM_2.5_	19	4–21 November 2016	310 ± 443	China, Xingping [79]
Housewife	PM_2.5_	19	January 2018	116 (32–224)	China, Xi’an, [80]
Highway toll station workers	PM_2.5_	16	March–May 2014	319.90	China, Tianjin [81]
Newsagent	PM	16	2013	5570	Iran, Tehran [82]
Seafarers	PM	32	July 2016	760–8400	Sweden [83]
Chinese kitchen worker	PM	16	4 September–1 November 2014	1794–12,108	China, Taiwan [84]

**Table 5:** Indoor concentrations (average and/or range, ng/m^3^) of PM-bound PAHs in several studies.

Place	Country, City	PM	PAHs	Periods	Concentration
Rural households	China, Laiyang [71]	PM	28	July	738 ± 321
Households	China, Hongkong [73]	PM_2.5_	26	2014–2016	3.0 (1.0–7.3)
	China, Xingping [79]	PM_2.5_	19	4–21 November 2016	211 ± 120
	China, Xi’an [80]	PM_2.5_	19	January 2018	92 (15–276)
	China, Beijing [88]	PM_2.5_	16	December 2014–February 2016	39.8
	Saudi Arabia, Jeddah [89]	PM_10_	13	-	18.5 ± 11.2
	Turkey, Bursa [90]	PM	16	July 2014– January 2015	22
	Spain, Madrid [91]	PM_10_	14	May 2017–April 2018	0.186
Infant room	China, Harbin [92]	PM	16	December 2013–March 2014	318 ± 314
University (dormitory)	China, Beijing [88]	PM_2.5_	16	December 2014–February 2016	34.1
	China, Wuhan [93]	PM_2.5_	16	December 2014–June 2015	31.3
University (laboratory)	China, Wuhan [93]	PM_2.5_	16	December 2014–June 2015	27.0
	China, Harbin [94]	PM_2.5_	16	January 2015	115
University (office)	China, Beijing [88]	PM_2.5_	16	December 2014–February 2016	32.1
	Saudi Arabia, Jeddah [89]	PM_10_	13	-	12.7 ± 5.1
	China, Wuhan [93]	PM_2.5_	16	December 2014–June 2015	32.4
	China, Harbin [94]	PM_2.5_	16	January 2015	96.6
Classroom	China, Beijing [95]	PM_2.5_	12	October 2016–March 2017	29.83
	Brazil, São Paulo [96]	PM	15	7–11 November 2016	0.45
	Poland, Warsaw [17]	PM_1_	16	April–June 2015	10.9
	Poland, Gliwice [17]	PM_1_	16	April–June 2015	21.6
	Portugal, Porto [97]	PM_2.5_	18	March–May 2014	5.03–23.6
Shopping malls	Pakistan, Islamabad [98]	PM_2.5_	16	February–April 2014	2.39 ± 1.45
Bakery	Italy, Bari [99]	PM_2.5_	7	7–19 April 2013	7.4
Hotels	Saudi Arabia, Jeddah [89]	PM_10_	13	-	6.3 ± 1.3
	China, Jinan [100]	PM_2.5_	19	January 2016	39.58–115.63
Public bars	Nigeria, Warri [101]	PM	16	-	43.43–155.11
Office building	China, Changchun [102]	PM_2.5_	16	April–October 2018 December 2017–April 2018	48.6 67.9
Fire station	Poland, North Poland [104]	PM_4_	15	September 2018	1882–5924
Ship	Sweden [83]	PM	32	July 2016	550–39,000
Chinese kitchen	China, Taiwan [84]	PM	16	4 September–1 November 2014	1648–5342

**Table 6:** Outdoor PM-bound PAHs concentrations (average and/or range, ng/m^3^) in some cities worldwide.

Country, City	PM	PAHs	Periods	Concentration
Oceania				
New Zealand, Auckland [108]	PM_2.5_	15	2016–2017	0.31 ± 0.19
Americas				
Mexico, South Mexico [67]	PM_2.5_	24	November 2016–March 2017	4.82 ± 1.97
Peru, Arequipa [109]	PM_2.5_ PM_10_	14	January–December 2018	7.4 ± 2.3 9.6 ± 3.9
Argentina, Cordoba [110]	PM_10_	14	August 2011–August 2013	4.5 ± 4.34
Brazil, Belo Horizonte [111]	PM_2.5_	16	May 2017–April 2018	1.68–6.24
Canada, Toronto [112]	PM_10_	17	August 2016–August 2017	10.2 ± 2.5
US, Washington [113]	PM_10_	19	April 2016–September 2018	0.84
Europe				
Spain, Coruña [114]	PM_10_	12	Januray–December 2017	7.56
Spain, South Spain [115]	PM_2.5_ PM_10_	16	July 2014–June 2015	23.0 26.2
Italy, Milan [116]	PM_2.5_	-	December 2018–February 2019 May–July 2019	72.8 ± 16.6 0.40 ± 0.07
Poland, Wadowice [117]	PM_10_	9	March 2017 August 2017	80.6 10.5
Cyprus, Nicosia [118]	PM_2.5_	50	January–March 2018	1.62
Czech Republic, Brno [119]	PM_1_	15	January–February 2017	20.7
Croatia, Zagreb [120]	PM_10_	10	December 2017–February 2018	25.4
Bosnia and Herzegovina, Sarajevo [120]	PM_10_	10	December 2017–February 2018	64.8
Russia, Moscow [121]	PM_10_	9	June–July 2018	1.32–7.68
Russia, St. Petersburg [121]	PM_10_	9	June–July 2018	1.71–6.30
Russia, Kazan [121]	PM_10_	9	June–July 2018	2.95–9.61
Africa				
Algeria, Algiers [122]	PM_10_	22	June–September 2016	7.47 ± 1.21
South Africa, Pretoria [123]	PM_2.5_	16	June–July 2016	4.11
Rwanda, Kigali [124]	PM_2.5_	15	May–June 2017	52.7
Asia				
China, Hongkong [73]	PM_2.5_	26	2014–2016	3. 9 (1.5–9.6)
China, Xi’an [127]	PM_2.5_ PM_10_	16	December 2016–December 2017	63.1 (14.3–266) 66.8 (9.69–349)
China, Shanghai [18]	PM_2.1_	9	July 2017 January 2018	1.36 ± 0.20 7.72 ± 3.33
China, Beijing [128]	PM_10_	15	January 2017	98.1 ± 48.2
China, Zhengzhou [128]	PM_10_	15	January 2017	77.9 ± 29.6
China, Guangzhou [129]	PM_2.5_	16	June–July 2016 November–December 2016	5.49 10.5
China, Taiyuan [129]	PM_2.5_	16	June–July 2016November–December 2016	29.5 197
China, Jinan [130]	PM_2.5_	18	March–December 2016	39.8 (8.18–246)
China, Shanxi [131]	PM_10_ PM_2.1_	17	January–February 2017	1056 ± 315 937 ± 294
China, Urumqi [132]	PM_2.5_	16	September 2017–September 2018	448
China, Chengdu [133]	PM_10_	16	March 2015–February 2016	82.0 ± 64.8
China, Changchun [134]	PM_2.5_	15	October–November 2016	81.4 ± 46.0
China, Harbin [135]	PM_2.5_	16	June 2017–May 2018	86.9
China, Lanzhou [136]	PM_2.5_	9	July 2017–October 2018	9.86
Japan, Kanazawa [137]	PM_2.5_	9	April 2017–February 2018	0.69
Japan, Chiba [138]	PM_2.5_	21	June 2016–October 2017	2.9
Japan, Kirishima [139]	PM_2.5_	9	November–December 2016	1.32 (0.36–2.90)
South Korea, Seoul [140]	PM_2.5_	14	January–December 2018	5.6 ± 7.9
South Korea, Gwangju [141]	PM_2.5_	17	October 2016–April 2017	1.04–29.5
Vietnam, Hanoi [142]	PM_10_	9	2016–2018	8.51
Singapore, Singapore [143]	PM_10_	16	May 2015–June 2016	0.68–5.97
Malaysia, Lumpur [144]	PM_2.5_	16	June 2015–May 2016	2.04 ± 0.28
Thailand, Chiang Mai [145]	PM_2.5_	8	February–April 2016	5.88 ± 1.97
Qatar, Doha [146]	PM_2.5_ PM_10_	36	May–December 2015	0.56 0.72
Lebanon, Beirut [147]	PM_2.5_	15	December 2018–October 2019	0.95
Mongolia, Ulaanbaatar [148]	PM_10_	15	January 2017 March 2017 September 2017	131–773 22.2–531 1.4–54.6
Pakistan, Islamabad [149]	PM_2.5_ PM_10_	16	January–September 2017	25.7 ± 12.0 40.1 ± 16.8
India, Jamshedpur [150]	PM_2.5_	16	December 2016–February 2017 March–May 2017	109 ± 18.2 81.1 ± 13.3
India, Delhi [151]	PM_2.5_	14	December 2016–December 2017	753 ± 252
India, Haryana [151]	PM_2.5_	14	December 2016–December 2017	259 ± 64.6
India, Uttar Pradesh [151]	PM_2.5_	14	December 2016–December 2017	535 ± 143
India, Pune [152]	PM_2.5_ PM_10_	16	March 2015–March 2016	342.4 ± 14.3 446.1 ± 25.6
Iran, Tehran [153]	PM_10_	16	February–March 2018	213 ± 145
Iran, Bushehr [154]	PM_2.5_	16	December 2016–September 2017	0.66–142.3

**Table 7:** Reference data of certain PAHs regarding their cancer risks based on BaP.

Species ^a^	1984 ^b^	1988 ^c^	1992 ^d^	1993 ^e^	1994 ^f^	1997 ^g^	1998 ^h^	1998 ^i^	2004 ^j^	2010 ^k^
US EPA 16 PAHs										
Acy			0.001		0.001					
Ace			0.001		0.001					
Flu			0.001		0.001					
Ant			0.01		0.01		0.0005			0
Phe			0.001			0.00064	0.005			0
FR			0.001		0.001		0.05			0.08
Pyr		0.081	0.001			0	0.001			0
BaA	0.013	0.145	0.1	0.1	0.1	0.014	0.005	0.1		0.2
Chr	0.001	0.0044	0.01	0.001	0.01	0.026	0.03	0.01		0.1
BbF	0.08	0.14	0.1	0.1	0.1	0.11	0.1	0.1	0.62	0.8
BkF	0.004	0.066	0.1	0.01	0.1	0.037	0.05	0.1	0.17	0.03
BaP	1	1	1	1	1	1	1	1	1	1
DBA	0.69	1.11	5	1	1	0.89	1.1			10
IDP	0.017	0.232	0.1	0.1	0.1	0.067	0.1	0.1		0.07
BgPe		0.022	0.01		0.01	0.012	0.02			0.009
Non-priority PAHs										
BcF										20
CcdP		0.023			0.1	0.012	0.02			0.4
BcP						0.023	0.023			
11H-BbcA										0.05
4H-CdefC										0.3
BjA										60
BeA										0.8
BlA										5
Per					0.001					
BjF		0.061			0.1	0.045	0.05	0.1	0.52	0.3
BeP		0.004			0.01	0	0.002			
Anth		0.32				0.28	0.3			0.4
DBacA					0.1					4
Cor					0.001					
NeP										0.3
DBaeF										0.9
DBalP							1	10		30
DBaeP							0.2	1		0.4
DBaiP						1.1	0.1	10	12	0.6
DBahP						1.2	1	10	11	0.9

^a^: Full name were shown in Table 2. ^b^: Chu, 1984 [165]; ^c^: Clement Associates, 1988 [166]; ^d^: Nisbet and Lagoy, 1992 [167]; ^e^: US EPA, 1993 [168]; ^f^: Malcolm and Dobson, 1994 [169]; ^g^: Muller et al., 1997 [170]; ^h^: Larsen and Larsen, 1998 [171]; ^i^: Collins et al., 1998 [172]; ^j^: Cal EPA, 2004 [173]; ^k^: US EPA, 2010 [160].

## References

[1] Lelieveld J., Evans J.S., Fnais M., Giannadaki D., Pozzer A. (2015). The contribution of outdoor air pollution sources to premature mortality on a global scale. Nature.

[2] Shah A.S., Langrish J.P., Nair H., McAllister D.A., Hunter A.L., Donaldson K., Newby D.E., Mills N.L. (2013). Global association of air pollution and heart failure: A systematic review and meta-analysis. Lancet.

[3] US EPA (2013). National Ambient Air Quality Standards for Particulate Matter.

[4] Esworthy R. (2013). Air Quality: EPA’s 2013 Changes to the Particulate Matter (PM) Standard.

[5] Kim K.-H., Kabir E., Kabir S. (2015). A review on the human health impact of airborne particulate matter. Environ. Int..

[6] WHO (2013). Health Effects of Particulate Matter. Policy Implications for Countries in Eastern Europe, Caucasus and Central Asia.

[7] Grivas G., Cheristanidis S., Chaloulakou A., Koutrakis P., Mihalopoulos N. (2018). Elemental composition and source apportionment of fine and coarse particles at traffic and urban background locations in Athens, Greece. Aerosol Air Qual. Res..

[8] Zhang L.L., Zhang X., Xing W.L., Zhou Q.Y., Yang L., Nakatsubo R., Wei Y.J., Bi J.R., Shima M., Toriba A. (2020). Natural aeolian dust particles have no substantial effect on atmospheric polycyclic aromatic hydrocarbons (PAHs): A laboratory study based on naphthalene. Environ. Pollut..

[9] Zhou Q.Y., Zhang L.L., Yang L., Zhang X., Xing W.L., Hu M., Chen B., Han C., Toriba A., Hayakawa K. (2021). Long-term variability of inorganic ions in TSP at a remote background site in Japan (Wajima) from 2005 to 2015. Chemosphere.

[10] Jiang N., Li Q., Su F., Wang Q., Yu X., Kang P., Zhang R., Tang X. (2018). Chemical characteristics and source apportionment of PM_2.5_ between heavily polluted days and other days in Zhengzhou, China. J. Environ. Sci..

[11] Zhang L.L., Morisaki H., Wei Y.J., Li Z.G., Yang L., Zhou Q.Y., Zhang X., Xing W.L., Hu M., Shima M. (2019). Characteristics of air pollutants inside and outside a primary school classroom in Beijing and respiratory health impact on children. Environ. Pollut..

[12] Zhang X., Zhang L.L., Yang L., Zhou Q.Y., Xing W.L., Toriba A., Hayakawa K., Wei Y.J., Tang N. (2020). Characteristics of polycyclic aromatic hydrocarbons (PAHs) and common air pollutants at Wajima, a remote background site in Japan. Int. J. Environ. Res. Public Health.

[13] Zhang H., Zhang L.L., Yang L., Zhou Q.Y., Zhang X., Xing W.L., Kazuichi H., Toriba A., Tang N. (2020). Impact of COVID-19 outbreak on the long-range transport of common air pollutants in KUWAMS. Chem. Pharm. Bull..

[14] Lee H., Honda Y., Hashizume M., Guo Y.L., Wu C.-F., Kan H., Jung K., Lim Y.-H., Yi S., Kim H. (2015). Short-term exposure to fine and coarse particles and mortality: A multicity time-series study in East Asia. Environ. Pollut..

[15] Karageorgou K., Manoli E., Kouras A., Samara C. (2020). Commuter exposure to particle-bound polycyclic aromatic hydrocarbons in Thessaloniki, Greece. Environ. Sci. Pollut. Res..

[16] Wang X., Banks A.P., He C., Drage D.S., Gallen C.L., Li Y., Li Q., Thai P.K., Mueller J.F. (2019). Polycyclic aromatic hydrocarbons, polychlorinated biphenyls and legacy and current pesticides in indoor environment in Australia–occurrence, sources and exposure risks. Sci. Total Environ..

[17] Rogula-Kozłowska W., Kozielska B., Majewski G., Rogula-Kopiec P., Mucha W., Kociszewska K. (2018). Submicron particle-bound polycyclic aromatic hydrocarbons in the Polish teaching rooms: Concentrations, origin and health hazard. J. Environ. Sci..

[18] Yang L., Zhang X., Xing W.L., Zhou Q.Y., Zhang L.L., Wu Q., Zhou Z.J., Chen R.J., Toriba A., Hayakawa K. (2021). Yearly variation in characteristics and health risk of polycyclic aromatic hydrocarbons and nitro-PAHs in urban shanghai from 2010 to 2018. J. Environ. Sci..

[19] Ai S., Qian Z.M., Guo Y., Yang Y., Rolling C.A., Liu E., Wu F., Lin H. (2019). Long-term exposure to ambient fine particles associated with asthma: A cross-sectional study among older adults in six low-and middle-income countries. Environ. Res..

[20] Beelen R., Hoek G., Raaschou-Nielsen O., Stafoggia M., Andersen Z.J., Weinmayr G., Hoffmann B., Wolf K., Samoli E., Fischer P.H. (2015). Natural-cause mortality and long-term exposure to particle components: An analysis of 19 European cohorts within the multi-center escape project. Environ. Health Perspect..

[21] Akhavan O., Bijanzad K., Mirsepah A. (2014). Synthesis of graphene from natural and industrial carbonaceous wastes. RSC Adv..

[22] Akhavan O., Ghaderi E., Akhavan A. (2012). Size-dependent genotoxicity of graphene nanoplatelets in human stem cells. Biomaterials.

[23] Akhavan O., Ghaderi E., Esfandiar A. (2011). Wrapping bacteria by graphene nanosheets for isolation from environment, reactivation by sonication, and inactivation by near-infrared irradiation. J. Phys. Chem. B.

[24] Hashemi E., Akhavan O., Shamsara M., Rahighi R., Esfandiar A., Tayefeh A.R. (2014). Cyto and genotoxicities of graphene oxide and reduced graphene oxide sheets on spermatozoa. RSC Adv..

[25] Akhavan O., Ghaderi E., Hashemi E., Akbari E. (2015). Dose-dependent effects of nanoscale graphene oxide on reproduction capability of mammals. Carbon.

[26] Niu Z., Liu F., Yu H., Wu S., Xiang H. (2021). Association between exposure to ambient air pollution and hospital admission, incidence, and mortality of stroke: An updated systematic review and meta-analysis of more than 23 million participants. Environ Health Prev. Med..

[27] IARC (2015). Outdoor Air Pollution.

[28] Stanaway J.D., Afshin A., Gakidou E., Lim S.S., Abate D., Abate K.H., Abbafati C., Abbasi N., Abbastabar H., Abd-Allah F. (2018). Global, regional, and national comparative risk assessment of 84 behavioural, environmental and occupational, and metabolic risks or clusters of risks for 195 countries and territories, 1990–2017: A systematic analysis for the global burden of disease study 2017. Lancet.

[29] WHO (2014). Who Guidelines for Indoor Air Quality: Household Fuel Combustion.

[30] WHO (2006). Air Quality Guidelines: Global Update 2005: Particulate Matter, Ozone, Nitrogen Dioxide and Sulfur Dioxide.

[31] INECC (2014). Mexico-Ambient Air Quality Standard.

[32] CAAQS (2020). Canadian Ambient Air Quality Standards (CAAQS) for Fine Particulate Matter (PM_2.5_) and Ozone. https://www.ccme.ca/files/current_priorities/aqms_elements/caaqs_and_azmf.pdf.

[33] Siciliano B., Dantas G., Silva C.M.D., Arbilla G. (2020). The updated Brazilian national air quality standards: A critical review. J. Braz. Chem. Soc..

[34] Díaz-Robles L., Saavedra H., Schiappacasse L., Cereceda-Balic F. (2011). The Air Quality in Chile.

[35] NEPM Australia (2016). Australia-National Air Quality Standards.

[36] Sato K., Ohara T. (2018). The status of PM_2.5_ pollution in Asia and direction toward solving the issue. Glob. Environ. Res..

[37] EU (2008). Directive 2008/50/EC of the European Parliament and of the council of 21 May 2008 on ambient air quality and cleaner air for Europe. Off. J. Eur. Union.

[38] Lisetskii F., Borovlev A. (2019). Monitoring of emission of particulate matter and air pollution using lidar in Belgorod, Russia. Aerosol Air Qual. Res..

[39] SANS (2005). South African National Standard (SANS). https://www.environment.gov.za/sites/default/files/docs/stateofair_executive_iaiquality_standardsonjectives.pdf.

[40] Zhang Y., Tao S. (2009). Global atmospheric emission inventory of polycyclic aromatic hydrocarbons (PAHs) for 2004. Atmos. Environ..

[41] Baek S., Field R., Goldstone M., Kirk P., Lester J., Perry R. (1991). A review of atmospheric polycyclic aromatic hydrocarbons: Sources, fate and behavior. Water. Air Soil. Pollut..

[42] Ambrose D., Ellender J., Sprake C., Townsend R. (1975). Thermodynamic properties of fluorine compounds. Part 15—Vapour pressures of the three tetrafluorobenzenes and 1,3,5-trichloro-2,4,6-trifluorobenzene. Faraday Transactions 1: Physical Chemistry in Condensed Phases. J. Chem. Soc..

[43] Coover M.P., Sims R.C. (1987). The effect of temperature on polycyclic aromatic hydrocarbon persistence in an unacclimated agricultural soil. Hazard. Waste Hazard. Mater..

[44] Goldfarb J.L., Suuberg E.M. (2008). Vapor pressures and enthalpies of sublimation of ten polycyclic aromatic hydrocarbons determined via the Knudsen effusion method. J. Chem. Eng. Data.

[45] Lee W.M.G., Tong H.C., Yeh S.Y. (1993). Partitioning model of PAHs between gaseous and particulate phases with consideration of reactivity of PAHs in an urban atmosphere. J. Environ. Sci. Health A.

[46] Lei Y.D., Chankalal R., Chan A., Wania F. (2002). Supercooled liquid vapor pressures of the polycyclic aromatic hydrocarbons. J. Chem. Eng. Data.

[47] PubChem National Library of Medicine, National Center of Biotechnology Information. https://pubchem.ncbi.nlm.nih.gov/.

[48] Yamasaki H., Kuwata K., Miyamoto H. (1982). Effects of ambient temperature on aspects of airborne polycyclic aromatic hydrocarbons. Environ. Sci. Technol..

[49] Hoyer H., Peperle W. (1958). Vapor pressure measurements on organic compounds and their sublimation heats. Z. Electrochem..

[50] Sonnefeld W., Zoller W., May W. (1983). Dynamic coupled-column liquid-chromatographic determination of ambient-temperature vapor pressures of polynuclear aromatic hydrocarbons. Anal. Chem..

[51] Hu H., Tian M., Zhang L., Yang F., Peng C., Chen Y., Shi G., Yao X., Jiang C., Wang J. (2019). Sources and gas-particle partitioning of atmospheric parent, oxygenated, and nitrated polycyclic aromatic hydrocarbons in a humid city in southwest China. Atmos. Environ..

[52] Ray D., Ghosh S.K., Raha S. (2019). Impacts of photochemical ageing on the half-lives and diagnostic ratio of polycyclic aromatic hydrocarbons intrinsic to PM_2.5_ collected from ‘real-world’ like combustion events of wood and rice straw burning. J. Hazard. Mater..

[53] Yang L., Tang N., Matsuki A., Takami A., Hatakeyama S., Kaneyasu N., Nagato E.G., Sato K., Yoshino A., Hayakawa K. (2018). A comparison of particulate-bound polycyclic aromatic hydrocarbons long-range transported from the Asian continent to the Noto peninsula and Fukue island, Japan. Asian J. Atmos. Environ..

[54] Yang L., Zhang L.L., Zhang H., Zhou Q.Y., Zhang X., Xing W.L., Takami A., Sato K., Shimizu A., Yoshino A. (2020). Comparative analysis of PM_2.5_-bound polycyclic aromatic hydrocarbons (PAHs), nitro-NPAHs (NPAHs) and water-soluble inorganic ions (WSIIs) at two background sites in japan. Int. J. Environ. Res. Public Health.

[55] Singh D.K., Kawamura K., Yanase A., Barrie L.A. (2017). Distributions of polycyclic aromatic hydrocarbons, aromatic ketones, carboxylic acids, and trace metals in arctic aerosols: Long-range atmospheric transport, photochemical degradation/production at polar sunrise. Environ. Sci. Technol..

[56] Tang N., Hakamata M., Sato K., Okada Y., Yang X., Tatematsu M., Toriba A., Kameda T., Hayakawa K. (2015). Atmospheric behaviors of polycyclic aromatic hydrocarbons at a Japanese remote background site, Noto Peninsula, from 2004 to 2014. Atmos. Environ..

[57] Zhang L.L., Yang L., Zhang H., Zhou Q.Y., Zhang X., Xing W.L., Toriba A., Hayakawa K., Tang N. (2020). Impact of the COVID-19 outbreak on the long-range transport of particulate PAHs in East Asia. Aerosol Air Qual. Res..

[58] WHO (2000). Part II Evaluation of Risks to Human Health, Chapter 5 Organic Pollutants. Air Quality Guidelines for Europe.

[59] Kim K.-H., Jahan S.A., Kabir E., Brown R.J. (2013). A review of airborne polycyclic aromatic hydrocarbons (PAHs) and their human health effects. Environ. Int..

[60] Armstrong B., Hutchinson E., Unwin J., Fletcher T. (2004). Lung cancer risk after exposure to polycyclic aromatic hydrocarbons: A review and meta-analysis. Environ. Health Perspect..

[61] Zheng Y., Jiang F., Feng S., Cai Z., Shen Y., Ying C., Wang X., Liu Q. (2021). Long-range transport of ozone across the eastern china seas: A case study in coastal cities in southeastern China. Sci. Total Environ..

[62] Bandowe B.A.M., Meusel H. (2017). Nitrated polycyclic aromatic hydrocarbons (nitro-PAHs) in the environment—A review. Sci. Total Environ..

[63] Taga R., Tang N., Hattori T., Tamura K., Sakai S., Toriba A., Kizu R., Hayakawa K. (2005). Direct-acting mutagenicity of extracts of coal burning-derived particulates and contribution of nitropolycyclic aromatic hydrocarbons. Mutat. Res..

[64] IARC (2013). Bitumens and Bitumen Emissions, and Some N- and S-Heterocyclic Polycyclic Aromatic Hydrocarbons.

[65] IARC (2013). Diesel and Gasoline Engine Exhausts and Some Nitroarenes.

[66] Li Y.J., Sun Y., Zhang Q., Li X., Li M., Zhou Z., Chan C.K. (2017). Real-time chemical characterization of atmospheric particulate matter in China: A review. Atmos. Environ..

[67] Amador-Muñoz O., Martínez-Domínguez Y., Gómez-Arroyo S., Peralta O. (2020). Current situation of polycyclic aromatic hydrocarbons (PAH) in PM_2.5_ in a receptor site in Mexico City and estimation of carcinogenic PAH by combining non-real-time and real-time measurement techniques. Sci. Total Environ..

[68] Cropper P.M., Overson D.K., Cary R.A., Eatough D.J., Chow J.C., Hansen J.C. (2017). Development of the GC-MS organic aerosol monitor (GC-MS OAM) for in-field detection of particulate organic compounds. Atmos. Environ..

[69] Zhang L.L., Yang L., Zhou Q.Y., Zhang X., Xing W.L., Wei Y.J., Hu M., Zhao L.X., Toriba A., Hayakawa K. (2020). Size distribution of particulate polycyclic aromatic hydrocarbons in fresh combustion smoke and ambient air: A review. J. Environ. Sci..

[70] WHO (2016). Ambient Air Pollution: A Global Assessment of Exposure and Burden of Disease.

[71] Zhang J., Liu W., Xu Y., Cai C., Liu Y., Tao S., Liu W. (2019). Distribution characteristics of and personal exposure with polycyclic aromatic hydrocarbons and particulate matter in indoor and outdoor air of rural households in northern China. Environ. Pollut..

[72] Orakij W., Chetiyanukornkul T., Chuesaard T., Kaganoi Y., Uozaki W., Homma C., Boongla Y., Tang N., Hayakawa K., Toriba A. (2017). Personal inhalation exposure to polycyclic aromatic hydrocarbons and their nitro-derivatives in rural residents in northern Thailand. Environ. Monit. Assess..

[73] Chen X.-C., Chuang H.-C., Ward T.J., Tian L., Cao J.-J., Ho S.S.-H., Lau N.-C., Hsiao T.-C., Yim S.H., Ho K.-F. (2020). Indoor, outdoor, and personal exposure to PM_2.5_ and their bioreactivity among healthy residents of Hong Kong. Environ. Res..

[74] Mu G., Fan L., Zhou Y., Liu Y., Ma J., Yang S., Wang B., Xiao L., Ye Z., Shi T. (2019). Personal exposure to PM_2.5_-bound polycyclic aromatic hydrocarbons and lung function alteration: Results of a panel study in china. Sci. Total Environ..

[75] Gatto M.P., Gariazzo C., Gordiani A., L’Episcopo N., Gherardi M. (2014). Children and elders exposure assessment to particle-bound polycyclic aromatic hydrocarbons (PAHs) in the city of Rome, Italy. Environ. Sci. Pollut. Res..

[76] Han J., Zhang N., Niu C., Han B., Bai Z. (2014). Personal exposure of children to particle-associated polycyclic aromatic hydrocarbons in Tianjin, China. Polycycl. Aromat. Compd..

[77] Che C., Li J., Dong F., Zhang C., Liu L., Sun X., Ma L., Qi H., Wang K. (2020). Seasonal characteristic composition of inorganic elements and polycyclic aromatic hydrocarbons in atmospheric fine particulate matter and bronchoalveolar lavage fluid of COPD patients in northeast China. Respir. Med..

[78] Svecova V., Topinka J., Solansky I., Rossner P., Sram R.J. (2013). Personal exposure to carcinogenic polycyclic aromatic hydrocarbons in the Czech Republic. J. Expo. Sci. Environ. Epidemiol..

[79] Li Y., Xu H., Wang J., Ho S.S.H., He K., Shen Z., Ning Z., Sun J., Li L., Lei R. (2019). Personal exposure to PM_2.5_-bound organic species from domestic solid fuel combustion in rural Guanzhong basin, China: Characteristics and health implication. Chemosphere.

[80] He K., Xu H., Feng R., Shen Z., Li Y., Zhang Y., Sun J., Zhang Q., Zhang T., Yang L. (2021). Characteristics of indoor and personal exposure to particulate organic compounds emitted from domestic solid fuel combustion in rural areas of northwest China. Atmos. Res..

[81] Zhao Y.-J., Shou Y.-P., Mao T.-Y., Guo L.-Q., Li P.-H., Yi X., Li Q.-Q., Shen L.-Z., Zuo H.-R., Wang J. (2018). PAHs exposure assessment for highway toll station workers through personal particulate sampling and urinary biomonitoring in Tianjin, China. Polycycl. Aromat. Compd..

[82] Rezaei F., Kakooei H., Ahmadkhaniha R., Azam K., Omidi L., Shahtaheri S.J. (2018). Inhalation exposure and health risks for newsagents exposed to atmospheric polycyclic aromatic hydrocarbons in Tehran, Iran. Urban Clim..

[83] Strandberg B., Österman C., Koca Akdeva H., Moldanová J., Langer S. (2020). The use of polyurethane foam (PUF) passive air samplers in exposure studies to PAHs in Swedish seafarers. Polycycl. Aromat. Compd..

[84] Wu M.-T., Lin P.-C., Pan C.-H., Peng C.-Y. (2019). Risk assessment of personal exposure to polycyclic aromatic hydrocarbons and aldehydes in three commercial cooking workplaces. Sci. Rep..

[85] Ghorbani M., Saleh H.N., Barjasteh-Askari F., Nasseri S., Davoudi M. (2020). The effect of gas versus charcoal open flames on the induction of polycyclic aromatic hydrocarbons in cooked meat: A systematic review and meta-analysis. J. Environ. Health Eng..

[86] Afé O.H.I., Douny C., Kpoclou Y.E., Igout A., Mahillon J., Anihouvi V., Hounhouigan D.J., Scippo M.-L. (2020). Insight about methods used for polycyclic aromatic hydrocarbons reduction in smoked or grilled fishery and meat products for future re-engineering: A systematic review. Food Chem. Toxicol..

[87] Zhang L.L., Morisaki H., Wei Y.J., Li Z.G., Yang L., Zhou Q.Y., Zhang X., Xing W.L., Hu M., Shima M. (2020). PM_2.5_-bound polycyclic aromatic hydrocarbons and nitro-polycyclic aromatic hydrocarbons inside and outside a primary school classroom in Beijing: Concentration, composition, and inhalation cancer risk. Sci. Total Environ..

[88] Chen Y., Li X., Zhu T., Han Y., Lv D. (2017). Pm2. 5-bound pahs in three indoor and one outdoor air in Beijing: Concentration, source and health risk assessment. Sci. Total Environ..

[89] Ali N. (2019). Polycyclic aromatic hydrocarbons (PAHs) in indoor air and dust samples of different saudi microenvironments; health and carcinogenic risk assessment for the general population. Sci. Total Environ..

[90] Esen F., Kayikci G. (2018). Polycyclic aromatic hydrocarbons in indoor and outdoor air in turkey: Estimations of sources and exposure. Environ. Forensics.

[91] Madruga D.G., Ubeda R.M., Terroba J.M., dos Santos S.G., García-Cambero J.P. (2019). Particle-associated polycyclic aromatic hydrocarbons in a representative urban location (indoor-outdoor) from south Europe: Assessment of potential sources and cancer risk to humans. Indoor Air.

[92] Li H.-L., Liu L.-Y., Zhang Z.-F., Ma W.-L., Sverko E., Zhang Z., Song W.-W., Sun Y., Li Y.-F. (2019). Semi-volatile organic compounds in infant homes: Levels, influence factors, partitioning, and implications for human exposure. Environ. Pollut..

[93] Wang G., Wang Y., Yin W., Xu T., Hu C., Cheng J., Hou J., He Z., Yuan J. (2020). Seasonal exposure to PM_2.5_-bound polycyclic aromatic hydrocarbons and estimated lifetime risk of cancer: A pilot study. Sci. Total Environ..

[94] Yury B., Zhang Z., Ding Y., Zheng Z., Wu B., Gao P., Jia J., Lin N., Feng Y. (2018). Distribution, inhalation and health risk of PM_2.5_ related pahs in indoor environments. Ecotoxicol. Environ. Saf..

[95] Ouyang R., Yang S., Xu L. (2020). Analysis and risk assessment of PM_2.5_-bound pahs in a comparison of indoor and outdoor environments in a middle school: A case study in Beijing, China. Atmosphere.

[96] Pereira D.C.A., Custódio D., de Andrade M.d.F., Alves C., de Castro Vasconcellos P. (2019). Air quality of an urban school in são Paulo city. Environ. Monit. Assess..

[97] Slezakova K., Oliveira M., Madureira J., Fernandes E.d.O., Delerue-Matos C., Morais S., Pereira M.d.C. (2017). Polycyclic aromatic hydrocarbons (PAH) in Portuguese educational settings: A comparison between preschools and elementary schools. J. Toxicol. Environ. A.

[98] Hamid N., Syed J.H., Junaid M., Mahmood A., Li J., Zhang G., Malik R.N. (2018). Elucidating the urban levels, sources and health risks of polycyclic aromatic hydrocarbons (PAHs) in Pakistan: Implications for changing energy demand. Sci. Total Environ..

[99] Ielpo P., Taurino M.R., Buccolieri R., Placentino C.M., Gallone F., Ancona V., Di Sabatino S. (2018). Polycyclic aromatic hydrocarbons in a bakery indoor air: Trends, dynamics, and dispersion. Environ. Sci. Pollut. Res..

[100] Li Y., Yang L., Chen X., Gao Y., Jiang P., Zhang J., Yu H., Wang W. (2017). PM2. 5-bound pahs in indoor and outdoor of hotels in urban and suburban of Jinan, China: Concentrations, sources, and health risk impacts. Aerosol Air Qual. Res..

[101] Adesina O.A., Nwogu A.S., Sonibare J.A. (2021). Indoor levels of polycyclic aromatic hydrocarbons (PAHs) from environment tobacco smoke of public bars. Ecotoxicol. Environ. Saf..

[102] Bai L., Chen W., He Z., Sun S., Qin J. (2020). Pollution characteristics, sources and health risk assessment of polycyclic aromatic hydrocarbons in pm2. 5 in an office building in northern areas, China. Sustain. Cities Soc..

[103] Arı A. (2020). A comprehensive study on gas and particle emissions from laser printers: Chemical composition and health risk assessment. Atmos. Pollut. Res..

[104] Rogula-Kozłowska W., Bralewska K., Rogula-Kopiec P., Makowski R., Majder-Łopatka M., Łukawski A., Brandyk A., Majewski G. (2020). Respirable particles and polycyclic aromatic hydrocarbons at two polish fire stations. Build. Environ..

[105] Yang L., Suzuki G., Zhang L., Zhou Q., Zhang X., Xing W., Shima M., Yoda Y., Nakatsubo R., Hiraki T. (2019). The characteristics of polycyclic aromatic hydrocarbons in different emission source areas in Shenyang, China. Int. J. Environ. Res. Public Health.

[106] Zhang L.L., Tokuda T., Yang L., Zhou Q.Y., Zhang X., Xing W.L., Wu Q., Zhou Z.J., Chen R.J., Kameda T. (2019). Characteristics and health risks of particulate polycyclic aromatic hydrocarbons and nitro-polycyclic aromatic hydrocarbons at urban and suburban elementary schools in Shanghai, China. Asian J. Atmos. Environ..

[107] Amarillo A.C., Carreras H. (2016). Quantifying the influence of meteorological variables on particle-bound PAHs in urban environments. Atmos. Pollut. Res..

[108] Kalisa E., Nagato E., Bizuru E., Lee K., Tang N., Pointing S., Hayakawa K., Archer S., Lacap-Bugler D. (2019). Pollution characteristics and risk assessment of ambient PM_2.5_-bound PAHs and NPAHs in typical Japanese and New Zealand cities and rural sites. Atmos. Pollut. Res..

[109] Valdivia A.E.L., Larico J.A.R., Peña J.S., Wannaz E.D. (2020). Health risk assessment of polycyclic aromatic hydrocarbons (PAHs) adsorbed in PM_2.5_ and PM_10_ in a region of Arequipa, Peru. Environ. Sci. Pollut. Res..

[110] Amarillo A.C., Mateos A.C., Carreras H. (2017). Source apportionment of PM_10_-bound polycyclic aromatic hydrocarbons by positive matrix factorization in Cordoba city, Argentina. Arch. Environ. Contam. Toxicol..

[111] Dos Santos R.R., de Lourdes Cardeal Z., Menezes H.C. (2020). Phase distribution of polycyclic aromatic hydrocarbons and their oxygenated and nitrated derivatives in the ambient air of a Brazilian urban area. Chemosphere.

[112] Jariyasopit N., Tung P., Su K., Halappanavar S., Evans G.J., Su Y., Khoomrung S., Harner T. (2019). Polycyclic aromatic compounds in urban air and associated inhalation cancer risks: A case study targeting distinct source sectors. Environ. Pollut..

[113] Kramer A.L., Campbell L., Donatuto J., Heidt M., Kile M., Simonich S.L.M. (2020). Impact of local and regional sources of PAHs on tribal reservation air quality in the US pacific northwest. Sci. Total Environ..

[114] Sánchez-Piñero J., Moreda-Piñeiro J., Concha-Graña E., Fernández-Amado M., Muniategui-Lorenzo S., López-Mahía P. (2020). Inhalation bioaccessibility estimation of polycyclic aromatic hydrocarbons from atmospheric particulate matter (PM_10_): Influence of PM_10_ composition and health risk assessment. Chemosphere.

[115] Pastor R.P., Salvador P., Alonso S.G., Alastuey A., dos Santos S.G., Querol X., Artíñano B. (2020). Characterization of organic aerosol at a rural site influenced by olive waste biomass burning. Chemosphere.

[116] Hakimzadeh M., Soleimanian E., Mousavi A., Borgini A., De Marco C., Ruprecht A.A., Sioutas C. (2020). The impact of biomass burning on the oxidative potential of PM_2.5_ in the metropolitan area of Milan. Atmos. Environ..

[117] Skiba A., Styszko K., Furman P., Dobrowolska N., Kistler M., Kasper-Giebl A., Zięba D. (2019). Polycyclic aromatic hydrocarbons (PAHs) associated with PM_10_ collected in Wadowice, south Poland. E3S Web Conf..

[118] Iakovides M., Iakovides G., Stephanou E.G. (2021). Atmospheric particle-bound polycyclic aromatic hydrocarbons, n-alkanes, hopanes, steranes and trace metals: PM_2.5_ source identification, individual and cumulative multi-pathway lifetime cancer risk assessment in the urban environment. Sci. Total Environ..

[119] Křůmal K., Mikuška P. (2020). Mass concentrations and lung cancer risk assessment of PAHs bound to pm1 aerosol in six industrial, urban and rural areas in the Czech Republic, central Europe. Atmos. Pollut. Res..

[120] Pehnec G., Jakovljević I., Godec R., Štrukil Z.S., Žero S., Huremović J., Džepina K. (2020). Carcinogenic organic content of particulate matter at urban locations with different pollution sources. Sci. Total Environ..

[121] Khalikov I., Korunov A. (2019). The content of high-molecular polycyclic aromatic hydrocarbons in urban air during FIFA World Cup 2018. Russ. J. Gen. Chem..

[122] Rabhi L., Lemou A., Cecinato A., Balducci C., Cherifi N., Ladji R., Yassaa N. (2018). Polycyclic aromatic hydrocarbons, phthalates, parabens and other environmental contaminants in dust and suspended particulates of Algiers, Algeria. Environ. Sci. Pollut. Res..

[123] Morakinyo O.M., Mukhola M.S., Mokgobu M.I. (2019). Concentration levels and carcinogenic and mutagenic risks of PM_2.5_-bound polycyclic aromatic hydrocarbons in an urban–industrial area in South Africa. Environ. Geochem. Health.

[124] Kalisa E., Nagato E.G., Bizuru E., Lee K.C., Tang N., Pointing S.B., Hayakawa K., Archer S.D., Lacap-Bugler D.C. (2018). Characterization and risk assessment of atmospheric PM_2.5_ and PM_10_ particulate-bound PAHs and NPAHs in Rwanda, Central-East Africa. Environ. Sci. Technol..

[125] Ofori S.A., Cobbina S.J., Doke D.A. (2020). The occurrence and levels of polycyclic aromatic hydrocarbons (PAHs) in African environments—A systematic review. Environ. Sci. Pollut. Res..

[126] Yan D., Wu S., Zhou S., Tong G., Li F., Wang Y., Li B. (2019). Characteristics, sources and health risk assessment of airborne particulate PAHs in Chinese cities: A review. Environ. Pollut..

[127] Wang L., Dong S., Liu M., Tao W., Xiao B., Zhang S., Zhang P., Li X. (2019). Polycyclic aromatic hydrocarbons in atmospheric PM_2.5_ and PM_10_ in the semi-arid city of Xi’an, northwest china: Seasonal variations, sources, health risks, and relationships with meteorological factors. Atmos. Res..

[128] Cao Z., Wang M., Shi S., Zhao Y., Chen X., Li C., Li Y., Wang H., Bao L., Cui X. (2020). Size-distribution-based assessment of human inhalation and dermal exposure to airborne parent, oxygenated and chlorinated PAHs during a regional heavy haze episode. Environ. Pollut..

[129] Song Y., Zhang Y., Li R., Chen W., Chung C.K.A., Cai Z. (2020). The cellular effects of PM_2.5_ collected in Chinese Taiyuan and Guangzhou and their associations with polycyclic aromatic hydrocarbons (PAHs), nitro-PAHs and hydroxy-PAHs. Ecotoxicol. Environ. Saf..

[130] Zhang Y., Yang L., Zhang X., Li J., Zhao T., Gao Y., Jiang P., Li Y., Chen X., Wang W. (2019). Characteristics of PM_2.5_-bound pahs at an urban site and a suburban site in Jinan in north china plain. Aerosol Air Qual. Res..

[131] Song W., Cao F., Lin Y.-C., Haque M.M., Wu X., Zhang Y., Zhang C., Xie F., Zhang Y.-L. (2019). Extremely high abundance of polycyclic aromatic hydrocarbons in aerosols from a typical coal-combustion rural site in china: Size distribution, source identification and cancer risk assessment. Atmos. Res..

[132] Wang W., Ding X., Turap Y., Tursun Y., Abulizi A., Wang X., Shao L., Talifu D., An J., Zhang X. (2020). Distribution, sources, risks, and vitro DNA oxidative damage of PM_2.5_-bound atmospheric polycyclic aromatic hydrocarbons in Urumqi, NW China. Sci. Total Environ..

[133] Yang J., Xu W., Cheng H. (2018). Seasonal variations and sources of airborne polycyclic aromatic hydrocarbons (PAHs) in Chengdu, China. Atmosphere.

[134] Wu X., Cao F., Haque M., Fan M.-Y., Zhang S.-C., Zhang Y.-L. (2020). Molecular composition and source apportionment of fine organic aerosols in northeast china. Atmos. Environ..

[135] Ma L., Li B., Liu Y., Sun X., Fu D., Sun S., Thapa S., Geng J., Qi H., Zhang A. (2020). Characterization, sources and risk assessment of pm2. 5-bound polycyclic aromatic hydrocarbons (PAHs) and nitrated PAHs (NPAHs) in Harbin, a cold city in northern China. J. Clean. Prod..

[136] Zhang L.L., Yang L., Bi J.R., Liu Y.Z., Toriba A., Hayakawa K., Nagao S., Tang N. (2021). Characteristics and unique sources of polycyclic aromatic hydrocarbons and nitro-polycyclic aromatic hydrocarbons inPM_2.5_ at a highland background site in northwestern China. Environ. Pollut..

[137] Xing W.L., Zhang L.L., Yang L., Zhou Q.Y., Zhang X., Toriba A., Hayakawa K., Tang N. (2020). Characteristics of PM_2.5_-bound polycyclic aromatic hydrocarbons and nitro-polycyclic aromatic hydrocarbons at a roadside air pollution monitoring station in Kanazawa, Japan. Int. J. Environ. Res. Public Health.

[138] Ichikawa Y., Watanabe T., Horimoto Y., Ishii K., Naito S. (2018). Measurements of 50 non-polar organic compounds including polycyclic aromatic hydrocarbons, n-alkanes and phthalate esters in fine particulate matter (PM_2.5_) in an industrial area of Chiba prefecture, Japan. Asian J. Atmos. Environ..

[139] Yang L., Zhou Q.Y., Zhang H., Zhang X., Xing W.L., Wang Y., Bai P., Yamauchi M., Chohji T., Zhang L.L. (2021). Atmospheric behaviour of polycyclic and nitro-polycyclic aromatic hydrocarbons and water-soluble inorganic ions in winter in kirishima, a typical japanese commercial city. Int. J. Environ. Res. Public Health.

[140] Kang M., Kim K., Choi N., Kim Y.P., Lee J.Y. (2020). Recent occurrence of pahs and n-alkanes in PM_2.5_ in Seoul, Korea and characteristics of their sources and toxicity. Int. J. Environ. Res. Public Health.

[141] Kim I., Lee K., Lee S., Kim S.D. (2019). Characteristics and health effects of PM_2.5_ emissions from various sources in Gwangju, South Korea. Sci. Total Environ..

[142] Pham C.-T., Boongla Y., Nghiem T.-D., Le H.-T., Tang N., Toriba A., Hayakawa K. (2019). Emission characteristics of polycyclic aromatic hydrocarbons and nitro-polycyclic aromatic hydrocarbons from open burning of rice straw in the north of Vietnam. Int. J. Environ. Res. Public Health.

[143] Urbančok D., Payne A.J., Webster R.D. (2017). Regional transport, source apportionment and health impact of PM_10_ bound polycyclic aromatic hydrocarbons in Singapore’s atmosphere. Environ. Pollut..

[144] Sulong N.A., Latif M.T., Sahani M., Khan M.F., Fadzil M.F., Tahir N.M., Mohamad N., Sakai N., Fujii Y., Othman M. (2019). Distribution, sources and potential health risks of polycyclic aromatic hydrocarbons (PAHs) in PM_2.5_ collected during different monsoon seasons and haze episode in Kuala Lumpur. Chemosphere.

[145] Thepnuan D., Yabueng N., Chantara S., Prapamontol T., Tsai Y.I. (2020). Simultaneous determination of carcinogenic PAHs and levoglucosan bound to PM_2.5_ for assessment of health risk and pollution sources during a smoke haze period. Chemosphere.

[146] Javed W., Iakovides M., Stephanou E.G., Wolfson J.M., Koutrakis P., Guo B. (2019). Concentrations of aliphatic and polycyclic aromatic hydrocarbons in ambient PM_2.5_ and PM_10_ particulates in Doha, Qatar. J. Air Waste Manag..

[147] Fadel M., Ledoux F., Farhat M., Kfoury A., Courcot D., Afif C. (2021). PM2. 5 characterization of primary and secondary organic aerosols in two urban-industrial areas in the East Mediterranean. J. Environ. Sci..

[148] Byambaa B., Yang L., Matsuki A., Nagato E.G., Gankhuyag K., Chuluunpurev B., Banzragch L., Chonokhuu S., Tang N., Hayakawa K. (2019). Sources and characteristics of polycyclic aromatic hydrocarbons in ambient total suspended particles in Ulaanbaatar city, Mongolia. Int. J. Environ. Res. Public Health.

[149] Mehmood T., Tianle Z., Ahmad I., Li X. (2020). Ambient PM_2.5_ and PM_10_ bound PAHs in Islamabad, Pakistan: Concentration, source and health risk assessment. Chemosphere.

[150] Kumar A., Ambade B., Sankar T.K., Sethi S.S., Kurwadkar S. (2020). Source identification and health risk assessment of atmospheric PM_2.5_-bound polycyclic aromatic hydrocarbons in Jamshedpur, India. Sustain. Cities Soc..

[151] Gadi R., Sharma S.K., Mandal T.K. (2019). Seasonal variation, source apportionment and source attributed health risk of fine carbonaceous aerosols over national capital region, India. Chemosphere.

[152] Roy R., Jan R., Gunjal G., Bhor R., Pai K., Satsangi P.G. (2019). Particulate matter bound polycyclic aromatic hydrocarbons: Toxicity and health risk assessment of exposed inhabitants. Atmos. Environ..

[153] Norouzian Baghani A., Bahmani Z., Sorooshian A., Farzadkia M., Nabizadeh R., Delikhoon M., Barkhordari A., Rezaei Kalantary R., Golbaz S., Kermani M. (2020). Characterization of polycyclic aromatic hydrocarbons associated with PM_10_ emitted from the largest composting facility in the Middle East. Toxin Rev..

[154] Akhbarizadeh R., Dobaradaran S., Torkmahalleh M.A., Saeedi R., Aibaghi R., Ghasemi F.F. (2020). Suspended fine particulate matter (PM_2.5_), microplastics (MPS), and polycyclic aromatic hydrocarbons (PAHs) in air: Their possible relationships and health implications. Environ. Res..

[155] Farahani V.J., Arhami M. (2020). Contribution of Iraqi and Syrian dust storms on particulate matter concentration during a dust storm episode in receptor cities: Case study of Tehran. Atmos. Environ..

[156] Tsiouri V., Kakosimos K.E., Kumar P. (2015). Concentrations, sources and exposure risks associated with particulate matter in the Middle East area—A review. Air Qual. Atmos. Health.

[157] Kim K.-H., Jahan S.A., Kabir E. (2013). A review on human health perspective of air pollution with respect to allergies and asthma. Environ. Int..

[158] Chang K.-F., Fang G.-C., Chen J.-C., Wu Y.-S. (2006). Atmospheric polycyclic aromatic hydrocarbons (PAHs) in Asia: A review from 1999 to 2004. Environ. Pollut..

[159] Unwin J., Cocker J., Scobbie E., Chambers H. (2006). An assessment of occupational exposure to polycyclic aromatic hydrocarbons in the UK. Ann. Occup. Hyg..

[160] US EPA (2010). Development of a Relative Potency Factor (RPF) Approach for Polycyclic Aromatic Hydrocarbon (PAH) Mixtures. External Review Draft.

[161] Rota M., Bosetti C., Boccia S., Boffetta P., La Vecchia C. (2014). Occupational exposures to polycyclic aromatic hydrocarbons and respiratory and urinary tract cancers: An updated systematic review and a meta-analysis to 2014. Arch. Toxicol..

[162] Petit P., Maître A., Persoons R., Bicout D.J. (2019). Lung cancer risk assessment for workers exposed to polycyclic aromatic hydrocarbons in various industries. Environ. Int..

[163] Poursafa P., Moosazadeh M., Abedini E., Hajizadeh Y., Mansourian M., Pourzamani H., Amin M.-M. (2017). A systematic review on the effects of polycyclic aromatic hydrocarbons on cardiometabolic impairment. Int. J. Prev. Med..

[164] Kalantary R.R., Jaffarzadeh N., Rezapour M., Arani M.H. (2020). Association between exposure to polycyclic aromatic hydrocarbons and attention deficit hyperactivity disorder in children: A systematic review and meta-analysis. Environ. Sci. Pollut. Res..

[165] Chu M.M. Evaluation and estimation of potential carcinogenic risks of polynuclear aromatic hydrocarbons. Proceedings of the Symposium of Polycyclic Aromatic Hydrocarbons in the Workplace, Pacific Rim Risk Conference.

[166] Clement Associates (1988). Comparative Potency Approach for Estimating the Cancer Risk Associated with Exposure to Mixtures of Polycyclic Aromatic Hydrocarbons.

[167] Nisbet I.C., Lagoy P.K. (1992). Toxic equivalency factors (TEFs) for polycyclic aromatic hydrocarbons (PAHs). Regul. Toxicol. Pharm..

[168] US EPA (1993). Provisional Guidance for Quantitative Risk Assessment of Polycyclic Aromatic Hydrocarbons.

[169] Malcolm H., Dobson S. (1994). The Calculation of an Environmental Assessment Level (EAL) for Atmospheric PAHs Using Relative Potencies.

[170] Muller P., Leece B., Raha D. (1997). Scientific Criteria Document for Multimedia Standards Development, Polycyclic Aromatic Hydrocarbons (PAH), Part 1: Hazard Identification and Dose-Response Assessment.

[171] Larsen J., Larsen P. (1998). Chemical carcinogens. Air Pollution and Health.

[172] Collins J., Brown J., Alexeeff G., Salmon A. (1998). Potency equivalency factors for some polycyclic aromatic hydrocarbons and polycyclic aromatic hydrocarbon derivatives. Regul. Toxicol. Pharmacol..

[173] CEPA (2004). No Significant Risk Levels (NSRLs) for the Proposition 65 Carcinogens Benzo[b]fluoranthene, Benzo[j]fluoranthene, Chrysene, Dibenzo[a,h]pyrene, Dibenzo[a,i]pyrene, and 5-Methylchrysene by the Oral Route.

[174] Kutralam-Muniasamy G., Pérez-Guevara F., Roy P.D., Elizalde-Martínez I., Shruti V. (2020). Impacts of the COVID-19 lockdown on air quality and its association with human mortality trends in megapolis Mexico City. Air Qual. Atmos. Health.

[175] Venter Z.S., Aunan K., Chowdhury S., Lelieveld J. (2021). Air pollution declines during COVID-19 lockdowns mitigate the global health burden. Environ. Res..

[176] Liu F., Wang M., Zheng M. (2021). Effects of COVID-19 lockdown on global air quality and health. Sci. Total Environ..

[177] Nie D., Shen F., Wang J., Ma X., Li Z., Ge P., Ou Y., Jiang Y., Chen M., Chen M. (2021). Changes of air quality and its associated health and economic burden in 31 provincial capital cities in China during COVID-19 pandemic. Atmos. Res..

[178] Durant J.L., Busby W.F., Lafleur A.L., Penman B.W., Crespi C.L. (1996). Human cell mutagenicity of oxygenated, nitrated and unsubstituted polycyclic aromatic hydrocarbons associated with urban aerosols. Mutat. Res..

